# Mapping the socio-ecological influences on child food literacy: a systematic scoping review

**DOI:** 10.1186/s13643-026-03159-0

**Published:** 2026-03-24

**Authors:** Sandhya Sahye-Pudaruth, Jess Haines, Alicia E. Martin, David W. L. Ma

**Affiliations:** 1 Department of Human Health and Nutritional Sciences, University of Guelph, 50 Stone Road East, Guelph, Ontario N1G 2W1 Canada; 2 Department of Family Relations and Applied Nutrition, University of Guelph, 50 Stone Road East, Guelph, Ontario N1G 2W1 Canada; 3 Department of Geography, Environment, and Geomatics, University of Guelph, 50 Stone Road East, Guelph, Ontario N1G 2W1 Canada

**Keywords:** Child food literacy, Socio-ecological model, Individual, Household, Community, Organizational

## Abstract

**Background:**

The prevalence of non-communicable diseases has increased in part due to a shift in food environments with more processed foods and low food skills among adults and children. Developing food literacy from early childhood is essential since food-related knowledge and skills develop throughout one’s life and are related to improved dietary intake. Food literacy is a multidimensional construct including food-related knowledge, skills, and self-efficacy used to access, prepare, and eat food. Given its multidimensionality, food literacy is shaped by a range of influences at various levels of the socio-ecological model for health (i.e., individual, household/interpersonal, community, and organizational). Therefore, we conducted a systematic scoping review to identify potential influences on children’s food literacy at the individual, household/interpersonal, community, and organizational levels.

**Methods:**

MEDLINE, Web of Science, and CINAHL databases were comprehensively searched. This scoping review was conducted in accordance with the PRISMA extension for Scoping Reviews and informed by Arksey and O’Malley’s framework.

**Results:**

Of 3082 titles and abstracts, 139 studies, i.e., randomized controlled trials, quasi/cluster-randomized trials, non-randomized controlled trials, qualitative, longitudinal and cross-sectional studies, were included in the scoping review. Findings show that most influences on child food literacy exist at individual (children’s individual characteristics), household (parents’ demographics, knowledge, and involvement) and organizational levels (school-based interventions, school environment) and highlight the lack of research examining influences at the community (community-based interventions, media campaigns, geographical locations) level. The representation of our results across the different levels of the socio-ecological model may be a starting point for interdisciplinary mapping to inform future research and help guide policy and program efforts to improve children’s food literacy.

**Systematic scoping review registration:**

OSF Registries (September 19, 2023) [https://osf.io/9p7n5].

**Supplementary Information:**

The online version contains supplementary material available at 10.1186/s13643-026-03159-0.

## Introduction

Marked shifts in global food systems have arisen resulting from changing food demands with urbanizing populations. This has contributed to shifts to a food system dominated by large multinational companies that control food production and distribution, and technological advancements resulting in increased food processing and marketing strategies [1]. Alongside these changes, we have seen a shift in food environments with more highly processed foods available and being consumed, which is characterized as the “Nutrition Transition” [2]. An increased consumption of these highly processed foods has been linked with higher prevalence of food-related chronic diseases such as type 2 diabetes, overweight and obesity, and cardiovascular diseases, which is primarily due to their high levels of saturated fats, sodium, and sugar [3–5]. Eating highly processed foods rather than cooking from scratch is now part of our societal norms, and this has led to “deskilling” and a generation that lacks the confidence and food skills required to cook from scratch [6–8].

Improving food literacy, especially from early childhood, has emerged as a way to address concerns related to the decrease in food skills [6] as there is evidence that food-related knowledge and skills develop across the lifespan and are related to improved dietary intake later in life [9]. Research further suggests that promoting healthy eating habits in children requires a need to move beyond the passive imparting of nutrition knowledge and towards more active, hands-on approaches such as learning to cook, prepare and plan meals [10]. Children with better cooking skills or those who participated in school-based interventions aimed at improving their cooking skills had higher intake of healthier foods such as fruit and vegetables, and whole grains [11–16] as well as improved anthropometrics measurement [17]. Despite this growing body of evidence, the inconsistency in the conceptualization and definition of food literacy limits our ability to synthesize its relationship with downstream health outcomes.

Researchers and practitioners are increasingly calling for broader conceptualizations of food literacy that consider the various influences within our complex food systems impacting food literacy and the ability to apply it (e.g., food systems and resulting environments) [18–20]. Cullen et al*.* define food literacy as:

the ability of an individual to understand food in a way that they develop a positive relationship with it, including food skills and practices across the lifespan in order to navigate, engage, and participate within a complex food system. It’s the ability to make decisions to support the achievement of personal health and a sustainable food system considering environmental, social, economic, cultural, and political components [18].

In addition to this broad definition, in practice, Martin and Massicotte (2021) have advocated for the incorporation of a food systems lens in the conceptualization of food literacy. This means not only focusing on individuals’ food and nutrition knowledge and skills, but also awareness of food systems, their influences on behavior, and how people can be empowered to change them [20]. Thus, current understanding of food literacy suggests it is a multidimensional concept that could be influenced at various levels of the socio-ecological model (SEM), i.e., individual, household/interpersonal, community, organizational and wider policy levels [21].

Little is known about the influences, i.e., family, institutions, policies, built environment, etc., on child food literacy within complex food systems. Without this information, we are at risk of developing policies and programs that lack coherence or recognition of the interconnectedness of food systems and related policies [22]. Thus, the overarching aim of this study was to identify socio-ecological influences associated with child food literacy. To achieve this aim, we conducted a scoping literature review, which is an appropriate method to “review” and “map” the current literature, in addition to identifying research gaps [23]. The specific objectives of this scoping review are 1) to identify potential influences on child food literacy using a socio-ecological perspective, and 2) to identify gaps in knowledge at the various socio-ecological levels. The SEM underscores the importance of examining influences at multiple levels of influence. Thus, this review will provide a holistic understanding of the complex set of factors influencing child food literacy across multiple levels of influence [24]. Results of this scoping review will help inform future research directions as well as programs and policies aimed at improving food literacy in children.

## Methods

### Protocol and registration

This scoping review was informed by the framework outlined by Arksey and O’Malley [23] and further refined by Levac et al. [25] and the Joanna Briggs Institute (JBI) [26]. Arksey and O’Malley’s [23] framework provided the foundational structure of the review while Levac et al. [25] strengthened the methodological rigor by refining the research question, search strategy, and study selection process. The JBI manual further provided a standardized process for data extraction and reporting using the Population, Concept and Context (PCC) framework [26]. The reported results are in accordance with the *PRISMA extension for Scoping Reviews* [27]. The review protocol was registered with *Open Science Framework* on September 19, 2023, and later updated to reflect two small changes to the study inclusion criteria (registration digital object identifier: https://osf.io/9p7n5).

### Eligibility criteria

The inclusion criteria were determined using the Population, Concept and Context framework (PCC) as outlined by JBI manual [26] (Table 1). The population of interest were children between the ages of 1.5 and 12 years or with an average age of 12 years or younger. Studies were deemed eligible if they were peer-reviewed, focused on influences (predictors and barriers) associated with food literacy in children at different levels of the SEM (individual, household/interpersonal, community, organizational, and wider policy level). The scoping review included randomized controlled, quasi-randomized/pre-post trials, longitudinal, cross-sectional, community-based participatory, mixed methods and qualitative studies without any geographical restrictions. Abstracts, case studies, conference proceedings, commentaries, editorials, letters to the editor, retracted studies, and studies conducted in children with malnutrition or nutrient deficiencies were excluded.

**Table 1:** Eligibility criteria

Study characteristic	Eligibility criteria
Population	Research conducted in healthy children between ages of 1.5 and 12 years or with an average age of 12 years or younger
Concept	Influences included predictors and barriers on food literacy in children
Context	Influences on child food literacy within the levels of the socio-ecological model (individual/household, community, organizational, and wider policy level)
Food literacy outcomes	Published literature on food literacy, food or/and nutrition literacy, food label/choice literacy, nutrition knowledge, cooking/culinary skills, food preparation skills, self-efficacy to cook or consume fruit and vegetables/healthy foods, food advertising literacy or indigenous food knowledge were included
Publication date	No restrictions
Publication type	Peer reviewed publication including randomized controlled trials (RCTs), quasi/cluster-randomized, pre/post-test designs, cluster non-RCTs, exploratory cluster RCTs, longitudinal, cross-sectionals, community-based participatory, mixed methods and qualitative studies
Geographical location	No restrictions
Language	Articles were restricted to English language only
Access	Available as full text online

### Search strategy

MEDLINE, Web of Science, and CINAHL databases were comprehensively searched on November 2nd, 2023. An updated search of these databases was conducted on November 19th, 2025. The search strategies used were based on previous literature [28], developed with a research librarian and discussed amongst co-authors. Search strategies were modified for the different databases used and limited to studies published in the English language. The search terms included “food*” combined with ((“litera*” OR skill*) AND (child* or preschool*)) further supplemented by a manual search (Supplementary Table S1). Reference lists of reviews, scoping reviews, systematic reviews, and meta-analyses relevant to food literacy and skills in children were verified to ensure that no relevant studies were missed.

### Study selection

**Fig. 1:**
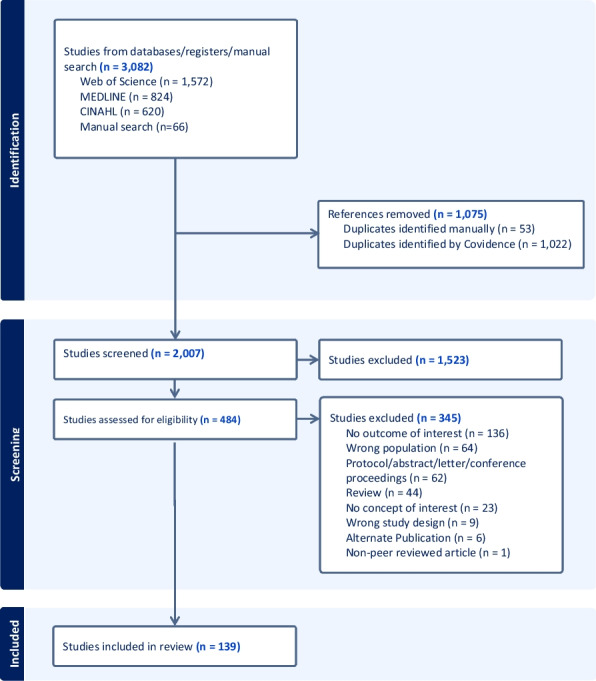
PRISMA flow diagram of systematic search of the literature

The search results from the three different databases were uploaded to EndNote and later exported to Covidence [29]. Covidence was used to remove all the duplicates, and one reviewer (SSP) screened the titles and abstracts of articles against the eligibility criteria (Table 1). The full texts for articles meeting the inclusion criteria were then retrieved and full text review was conducted by a single reviewer (SSP). To mitigate potential bias, any uncertainties about study selection and eligibility were discussed with two other reviewers (AM and JH) and resolved through consensus. Figure 1 presents a flow diagram of articles screened, assessed for eligibility and included in the review.

### Data extraction and analysis

Data extraction was independently completed by one single reviewer (SSP). To enhance the accuracy and minimize potential bias, the reviewer engaged in iterative discussions with a second reviewer (AM) and the broader research team to resolve uncertainties, ensure consistency and alignment with the predefined data charting. The data extraction framework was developed based on the information retrieved from Arksey and O’Malley’s framework [23] as well as JBI’s manual for charting the data [26]. The study characteristics extracted included information on author, year of publication, country of origin, study design and setting, target population, study aims, intervention details (where applicable), influences on child food literacy and their corresponding level of SEM, the outcomes, measures of food literacy (including their validation status), and the variables controlled for in each study (Supplementary Table S2). Risk of bias is not reported since we are scoping the literature around factors influencing child food literacy without assessing any bias.

### Data synthesis

The SEM was used to organize and map the influences on child food literacy of the included studies. SEM is a conceptual framework used to understand the complex interplay between multiple levels of factors such as individual, household/interpersonal, community, organizational, and wider policy level [30]. The SEM emphasizes the dynamic interactions between its different levels and the importance of the social environment for behavior change. It recognizes that individual behaviors are influenced by a complex interplay of factors at multiple levels of influence and not solely determined by individual choice [30, 31]. As data were extracted, one reviewer (SSP) organized data into their respective socio-ecological levels. For example, studies that examined individual level factors, such as age and sex, were categorized under the individual level; those examining family and peer factors were categorized under the household/interpersonal level; studies examining community level factors such as the neighborhood and community-based interventions were categorized under the community level; school-based interventions conducted in school settings and those targeting the school environments were categorized as organizational. Some studies also reported influences on child food literacy at more than one socio-ecological level in which case they were categorized at more than one level. The most common level that overlapped with each other were the individual and organizational levels (Supplementary Table S2).

## Results

### Search results

A total of 3082 studies were identified from the electronic databases and manual search. After removing the duplicates and screening the titles and abstracts, 484 studies were eligible for full text review. A final sample of 139 studies met the inclusion criteria and were included in the scoping review (Fig. 1).

### Study characteristics

The study characteristics of the 139 studies were presented in Supplementary Table S2. The majority of the studies were of quasi-experimental or pre-post intervention designs (*n* = 73, 52.5%) and the rest were of cross-sectional (*n* = 19, 13.7%); randomized or cluster randomized controlled trials (*n* = 19, 13.7%); mixed methods (*n* = 9, 6.5%); qualitative (*n* = 8, 5.8%); and longitudinal (*n* = 3, 2.2%); cluster non-randomized (*n* = 1, 0.7%); community-based participatory research (*n *= 1, 0.7%); controlled, non-randomized (*n* = 1, 0.7%); exploratory cluster randomized controlled (*n* = 1, 0.7%); historical design control (*n* = 1, 0.7%); multi-phase study design with study tool development and validation (*n* = 1, 0.7%); randomized block design (*n* = 1, 0.7%); single-blinded, parallel, randomized controlled pilot (*n* = 1, 0.7%). Most of the included studies were conducted in the USA (*n* = 64, 46%), followed by Canada (*n* = 15, 10.8%), Australia (*n* = 10, 7.2%), UK (*n* = 9, 6.5%), and Italy (*n* = 6, 4.3%). There were 110 studies conducted in school settings (79.1%), 22 studies were community-based (15.8%), 5 studies were conducted in home-based settings (3.6%), 1 study was conducted in both lab and home settings (0.7%), and 1 study was conducted in a day care setting (0.7%). Sample size ranges from 7 to 11,384 children, with most studies based on a sample of $\le$ 300 children (*n* = 88; 63.3%). The majority of the studies (*n* = 89; 64%) were published within the last decade highlighting increasing interest in the concept of child food literacy. Across the included studies, there was considerable variability in whether and how the potential confounders were measured or adjusted for (Supplementary Table S3).

### Measurement of child food literacy outcomes

The most frequently measured aspects of child food literacy across the eligible studies were nutrition knowledge, cooking skills, food label literacy, food preparation skills, and self-efficacy to cook. Various measurement tools were utilized to measure these aspects of child food literacy across the included studies with surveys being the most commonly used. The tools to measure child food literacy were either developed for a specific study or adapted from existing and previously validated ones such as Tool for Food Literacy Assessment in Children (TFLAC) [32] and Knowledge, Attitudes, and Behaviours (KAB) [33], but the majority lacked reported validation. Out of 139 included studies, only 41 studies (29.5%) used validated tools to measure child food literacy. Sixty-two percent of the tools (*n* = 86) demonstrated limited psychometric properties or reported no validation. The remaining studies (*n* = 12; 8.6%) did not use any formal measurement tools due to their qualitative design (Supplementary Table S3).

### Overview of the evidence

**Fig. 2:**
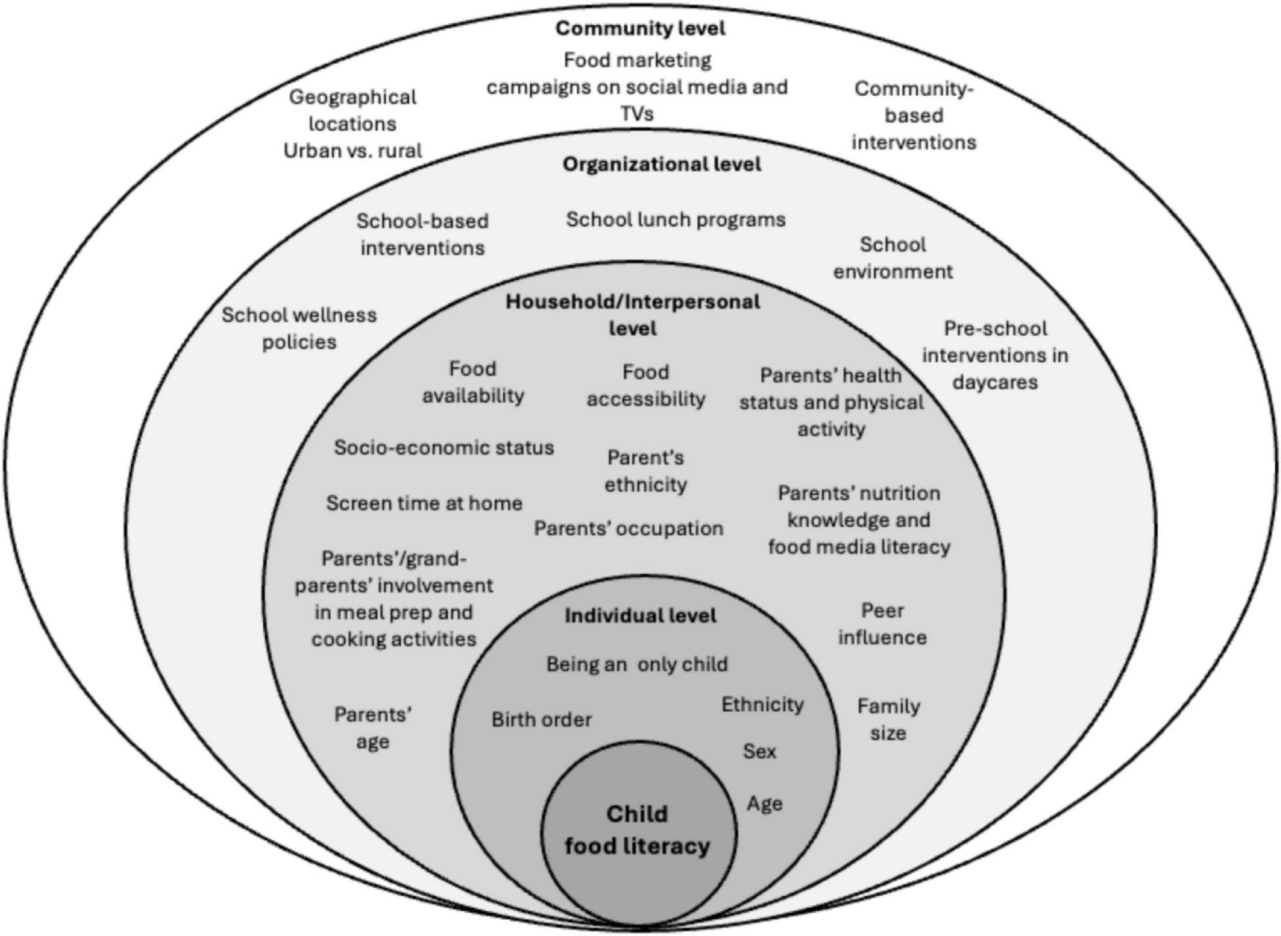
A model of predictors showing from a socio-ecological perspective [30] the influences on child food literacy. Influences at the individual level were shaped by factors at the other levels, including household, interpersonal, community, and organizational levels

Studies assessed various influences on child food literacy. Using the SEM [30] for mapping, the potential influences were nested under the four different levels (e.g., individual, household/interpersonal, community and organizational) as illustrated in the model of predictors below (Fig. 2). A summary of the influences organized by the different levels of the SEM is provided below. However, it is important to note that no influences on child food literacy were identified at the policy level and thus are not discussed here. All included studies (*n* = 139) reported influences on child food literacy at the individual level (Supplementary Table S3). While several of the influences on child food literacy were at the individual level (children’s individual characteristics), these were often shaped by factors at the other levels, including household and interpersonal (parents’ demographics, knowledge and involvement, and peers’ influence), community (geographical locations (rural versus urban), media campaigns and community-based interventions), and organizational (school wellness policies, school-based interventions, school lunch programs, broader school environment, day care interventions) (Fig. 2). For example, parental food literacy at the household level may be shaped by factors at the organizational and community levels, which may in turn influence child food literacy at the individual level. This is reflected in the fact that of the 139 articles included in the review, 137 included influences at multiple socio-ecological levels: individual/organizational (*n* = 65); individual/household/organizational (*n* = 23); individual/household (*n* = 22); individual/community (*n* = 14); individual/household/community (*n* = 10); individual/household/interpersonal (*n *= 1); individual/household/interpersonal/organizational (*n* = 1); individual/household/community/organizational (*n* = 1).

#### The individual level

Nineteen articles [34–52] reported on children’s characteristics such as age, sex, ethnicity, and birth order or being an only child influencing child food literacy at the individual level.

Out of the 8 studies that reported findings on age, most of them suggested that older children had greater food or nutrition literacy [42, 50, 51] nutrition knowledge [41, 51], food preparation skills [36, 39], and were more knowledgeable in categorizing foods between “healthy” and “unhealthy” [37]. On the other hand, Colley et al. (2022) reported no association between children’s age and nutrition knowledge [46].

Child sex was also associated with child food literacy at the individual level, but with mixed findings. Girls were reported to have better food or nutrition literacy [42, 43, 45, 50, 51] and food choice literacy (described as the ability to make informed decisions about food [53]), while boys had a higher level of critical food and nutrition literacy [38], meaning they have a better ability to critically evaluate nutritional information [38], and were more likely to help prepare meals than girls [40]. Additionally, girls were also reported to have higher nutrition knowledge [43, 45–47] and cooking self-efficacy than boys [52]. In contrast, an article by Pirouznia (2001) observed no difference in nutrition knowledge between girls and boys in 6th grade, but found that girls in the 7th and 8th grades had a significantly higher nutrition knowledge than boys [34]. Moreover, two additional studies reported that post-intervention, boys significantly improved their cooking skills and food and nutrition knowledge compared to girls [35, 49]. This particular finding was attributed to the fact that girls in both studies had a higher level of cooking skills and food knowledge at baseline, leaving less room for improving their food literacy [35, 49]. Two other studies further reported no difference in food literacy between girls and boys in terms of food preparation [36] and nutrition literacy [48].

Race/ethnicity was further seen as another factor associated with child food literacy. White children had higher self-efficacy for cooking [36] and possessed higher nutrition knowledge [46] than other ethnic minorities. An additional study reported that children’s birth order (being a first born) was associated with food literacy [44].

#### The household level

A total of 57 (41%) studies examined the influences on child food literacy at the household level, including the influence of parents’ demographics, characteristics, nutrition knowledge, food skills and education, early involvement of children in cooking and meal preparation activities, knowledge of food media literacy, as well as the influence of the home environment on child food literacy (Supplementary Table S3).

##### Parents’ demographics or characteristics

Fourteen studies [38, 40, 42, 44–47, 50, 54–59] reported findings on parents’ demographics or characteristics, which included parental socio-economic status (SES), occupation, education, age, ethnicity, health status/physical activity level/nutrition knowledge, and family size. Eight studies reporting on parental SES consistently mentioned that higher parental SES was directly associated with an improvement in nutrition knowledge, food and nutrition literacy, and self-efficacy to prepare and choose healthy foods [38, 45, 46, 50, 55–57, 59]. Parents with higher SES have the privilege to enroll their children in private schools, which was associated with better child food literacy [59]. On the other hand, 2 other studies reported that children from lower SES possessed more food skills and knowledge related to traditional foods [42] and boys from insecure households were 65% more likely to help with food preparation and cooking than boys in food secure households [40].

Parental education was assessed in 8 studies with consistent results showing that higher parental (both maternal and paternal) education was associated with a higher level of food and nutrition literacy or nutrition knowledge in children [38, 45–47, 50, 55, 58, 59]. Parents’ age and ethnicity, and family size were also associated with child food literacy in 2 studies published in Iran [38, 59]. Children whose parents were older (fathers: aged between 41 and 45 years old and mothers: aged between 36 and 40 years old) had higher nutrition knowledge and food choice literacy [38] while on the other hand, another study by Doustmohammadian 2022 observed that as mothers’ age increases, critical food and nutrition literacy decreases in children [59]. Alternatively, children whose parents were ethnic minorities were reported as possessing low nutrition knowledge and critical food and nutrition literacy [38, 59]. Additionally, children from small family sizes had “adequate” levels of food and nutrition literacy due to socio-economic factors such as higher parental education and SES [59].

Several other parental factors such as parental health status [44], nutrition knowledge [47, 55], physical activity level [42], food skills [60] and education [61] were identified as important influences on child food literacy. Higher paternal hypertension was associated with lower nutrition literacy in children while parental health status such as paternal diabetes and maternal dyslipidemia was associated with higher levels of nutrition literacy in children [44]. Parents’ nutrition knowledge and food education are strong predictors of children’s food literacy indicating that parents with better nutrition knowledge or food education had children with greater nutrition knowledge [47, 55], food systems knowledge [54], and food literacy [61]. Children’s food literacy also seemed to be associated with mothers’ physical activity, that is, a higher level of physical activity was related to higher food literacy in children [58]. On the other hand, another study reported that although parents possessed relatively good food skills, there was no significant association between their food skills and children’s cooking skills [60].

##### Parental involvement in meal preparation and cooking activities, and nutrition education

Other than parental demographics or characteristics mentioned above, the involvement of children in meal preparation and cooking activities with parents or grandparents/caregivers in home, community, or school-based settings was associated with child food literacy (Supplementary Table S3). Parents engaging in cooking or meal preparation activities with their children were associated with better cooking and preparation skills [62–69], nutrition knowledge [11, 56, 66, 70–74], menu planning [73, 74], ancestral food knowledge [75], food and nutrition literacy [76], food choice literacy [77], self-efficacy to cook and prepare foods [11, 36, 56, 78], consume/cook fruit and vegetables [71], and select healthy foods [56, 79] in children. Additionally, 3 studies reported on the impact of intergenerational influence, that of grandparents on children’s cooking skills [65, 80] and food and nutrition literacy [45].

Furthermore, studies targeting not only children, but also their parents’ food literacy and nutrition knowledge as a means to potentially influence children’s food literacy were identified (Supplementary Table S3). Parents involved interacted with their children in nutrition education sessions or were provided with printed newsletters or sent reinforcement messages which reported an improvement in their children’s nutrition knowledge [72, 81–89], food label literacy [81, 86], food preparation and cooking skills [82, 90, 91], kitchen safety knowledge [81], self-efficacy to choose healthy foods [87], and making sustainable food choices [90]. But exposure to nutrition interventions with reinforcement messages to parents was also associated with no improvement in children’s food label literacy [92] and nutrition knowledge [93]. In addition to parents and grandparents, peers were also identified as playing a role in children’s food literacy. Research has demonstrated that they were associated with children’s nutritional knowledge [47, 84].

##### Household food access

Two articles looked at the association between food accessibility and child food literacy [45, 56]. Lack of access to food was associated with lower self-efficacy to prepare and choose healthy foods [56] and food and nutrition literacy in children [45].

##### Screen time at home

Two studies focused on the use of media such as watching TV, playing online games, and using social media and its association with child food literacy. They reported that lower screen time was associated with higher nutrition literacy in children [41, 48].

##### Parental food media literacy

Three studies that analyzed the effects of food and advertising literacy training in both parents and children reported that exposure to food and advertising literacy improved parents’ awareness of media exposure and decreased children’s susceptibility to food marketing messages [94–96]. Another study reported food advertising literacy improved when training was accompanied by parental support [97]. Media literacy training in children further helped them to critically understand food marketing messages and assess the nutritional content of packaged foods, therefore potentially limiting their susceptibility to unhealthy food decisions. 

#### The organizational level

There were 90 (64.7%) studies that examined organizational influences on child food literacy. Of these, 93.3% (*n* = 84) were multi-level studies that assessed the influence of interventions in schools (*n* = 83) or day care (*n *= 1) on children’s food and nutrition knowledge, attitudes and skills, as well as changes to the broader school environment (Supplementary Table S3). The remaining 6 (4.3%) studies, also conducted in school settings, were qualitative in nature and provided insights on the different factors influencing food literacy in children.

##### Student-level interventions that focused on changing food and nutrition knowledge, attitudes and skills

School-based interventions designed to improve child food literacy were presented in 73 studies. These interventions incorporated interactive components such as cooking, preparing healthy or plant-based foods, taste testing, gardening, growing and harvesting foods, visits to local farms, and virtual games. Others were more educational with a focus on food labeling, nutrition and physical activity education, food waste, and media literacy training, but all studies aimed to improve children’s food literacy overall. Children exposed to these activities were reported to have improved food literacy [43, 98], food label literacy [86, 99–103], food preparation and cooking skills [43, 49, 63, 66, 104–110], cooking confidence [15], self-efficacy to prepare and cook food [71, 82, 91, 111–116], food systems knowledge [54, 117], food/kitchen hygiene and storage knowledge [43, 107, 118], menu planning skills [17, 73, 74], self-efficacy to eat fruit and vegetables [13, 71, 113], gardening skills [109, 113], food advertising and media literacy [101, 119], food knowledge [120], food waste behaviors [110], and nutrition knowledge [33, 43, 49, 66, 70, 71, 73, 74, 82, 85, 86, 88, 89, 98, 99, 102, 103, 107, 108, 110, 112, 117, 119, 121–147]. On the contrary, although the above interventions reported improvements in overall food literacy, some did not, as children exposed to school-based interventions showed no improvement in self-efficacy to cook or cooking skills [138, 145, 148], eat fruits and vegetables, and garden [145], food label literacy [92, 139], food systems knowledge [148], and nutrition knowledge [93, 148–150].

##### School-level interventions to change students’ food literacy, food offerings and the school food culture

Also at the organizational level, 11 studies (Supplementary Table S3) reported that modifying the school food environment alongside a curriculum on healthy eating and nutrition education classes was associated with child food literacy as schools were identified as key settings for the development of early food literacy in children [151]. School-based interventions that combined both nutrition education and meal prep activities together with changes to the school environment such as providing more fresh produce or installing salad bars to provide fresh seasonal fruits and vegetables for lunch reported an improvement in the children’s nutrition knowledge and vegetable identification [72, 83]. Further modification of school food environments by implementing healthy food competitions, food and nutrition festivals, healthy nutrition painting contests among children, distributing healthy snacks at the school canteen at an affordable price or free of charge, and by initiating a School Food and Nutrition Committee was reported to be associated with improved food choice literacy in children [77].

A school lunch program providing students with low-fat and sugar alternatives at a lower cost together with a curriculum component on healthy eating, physical activity and diabetes education in a First Nations community was reported to improve nutrition knowledge in children [84]. Another school that promoted weekly cooking sessions on plant-based diets and included a recipe from the cooking workshops to an existing school lunch program in order to make whole grains and vegetables more accessible to students observed an improvement in nutrition knowledge and self-efficacy to cook [11]. Incorporating locally grown foods in school lunch menus [152] or a program aimed to change the whole school food environment by increasing exposure to local foods with enhanced food presentations in school cafeterias [153] also improved nutrition knowledge, food label literacy, agri-food knowledge, and self-efficacy to choose healthy foods [152, 153]. Moreover, a school program that promoted healthier dietary choices among students by using the traffic light system and introducing cues in the school cafeteria found an improvement in their overall food literacy and nutrition knowledge [154].

Further changes to school menus to supply 35% of the Recommended Daily Allowances (RDA) for energy, protein, calcium, and iron together with nutrition and meal preparation activities reported to be associated with nutrition knowledge, food preparation skills, food label literacy, and kitchen safety knowledge [81]. Additionally, the creation of school wellness policies and policy recommendations within the school community involving school-based physical activity and healthy dietary changes together with nutrition education classes was associated with nutrition knowledge and self-efficacy for choosing healthy foods [87]. On the other hand, a school-based intervention that included a food literacy curriculum and a centrally procured snack program to increase fruit and vegetable intake reported no change in nutrition knowledge [155].

##### A daycare intervention that aimed to improve food literacy in preschool children

Among preschool children in a day care setting, exposure to a nutrition education program aiming to identify and prepare nutritious snacks was associated with children’s nutrition knowledge [156].

##### Qualitative studies conducted in school settings

Six qualitative studies conducted in school settings provided more perspectives on the factors influencing food literacy in children (Supplementary Table 3). Four qualitative studies exploring children’s thoughts and perspectives on their experiences with school-based interventions reported that exposure to these interventions enhanced their nutrition knowledge, food preparation and cooking skills, self-efficacy to choose and prepare foods, and gardening skills [56, 104, 157, 158]. A study that explored children’s perceptions on categorizing healthy versus unhealthy foods reported that children were able to do so due to exposure to nutrition education in the schools [37]. Another qualitative study found that providing children with opportunities to understand the domain of food and food systems in schools may improve their knowledge of food systems [54].

#### The community level

A total of 25 studies examined community-level influences on child food literacy, of which 22 (88%) were community-based interventions aimed to improve food literacy in children. Three other studies were of cross-sectional (*n* = 2) and qualitative (*n* = 1) designs that assessed the influence of geographical locations (urban versus rural) and community-level food marketing campaigns on child food literacy.

##### Interventions in community-based settings

Interventions conducted in community-based settings (*n* = 22) aiming to positively impact children’s food literacy included components such as nutrition education, food preparation, cooking workshops, food tasting, gardening activities, and media literacy and food marketing training (Supplementary Table S3). These community-based interventions reported an improvement in nutrition knowledge [35, 159–166], food preparation and cooking skills [39, 62, 65, 80, 159, 163–166], cooking self-efficacy/confidence [52, 78, 159, 160, 165], self-efficacy to eat and cook fruit and vegetables [167], and choose healthy foods [95], gardening skills [168], planning a meal [169], media, food marketing and advertising literacy [94, 96], food label literacy [95, 96], ancestral food knowledge [75], and food safety practices [164]. On the contrary, 2 studies reported no change in children’s nutrition knowledge [168] and cooking skills [161] after exposure to community-based interventions.

##### Geographical locations (urban versus rural)

The two cross-sectional studies that examined how geographical locations (urban versus rural) were associated with child food literacy [45, 46] had mixed findings. Those living in Canadian rural areas and “urban small towns” had higher nutritional knowledge than those living in urban areas [46] whereas Liu et al*.* (2021) reported that children living in Chinese urban neighborhoods were reported to have higher food and nutrition literacy than children living in rural areas [45].

##### Food marketing campaigns

A single qualitative study found that mass media providing nutritional messages, social media marketing campaigns, advertisements on TV, reality cooking shows, and use of mobile apps to learn about healthy eating may be factors that were associated with child food literacy at the community level [56] For example, children mentioned obtaining health promotional messages by governmental agencies through media campaigns or from cooking shows hosted by famous chefs [56].

## Discussion

### Synthesis of evidence

In this scoping review, we identified and summarized evidence from 139 studies that not only assessed the socio-ecological influences on child food literacy but also highlighted the complexity and multidimensionality of factors influencing child food literacy. Results show that most research examining influences on child food literacy occurs at the individual and organizational levels, highlighting the lack of studies at the community and policy levels. All the included studies (*n* = 139) in this scoping review included individual level influences on child food literacy. This is not surprising as most food literacy research has focused on a narrow definition of food literacy limited to possessing food and nutrition related skills and knowledge such as the ability to cook and understand nutrition labels at the individual level [170]. This approach to food literacy is limited as it presents food literacy as a construct aimed solely at improving individualized food choices and dietary behavior, rather than also improving knowledge to address the broader socio-ecologic factors that produce unhealthy food environments and enable or inhibit said choices [171, 172]. Moreover, targeting individual knowledge and skills instead of broader context is seen as being more modifiable and closely related to individual health by researchers, which aligns with other research findings [173, 174]. However, since child food literacy is “complex and multifaceted” [170], the factors influencing it are interconnected with other levels in the socio-ecological model and there is a need to target the broader environment beyond the individual, while also empowering individuals to change the systems and environments that constrain “choice” [171].

Of the included studies, 42% of them included factors influencing child food literacy at the household level. This likely reflects the perception that parents are mainly responsible for promoting healthy eating behaviors in children, which can last until adulthood [9, 175, 176]. Given their role as their children’s “dietary gatekeepers,” parents play an important role in the development of their children’s food literacy [177]. In general, parental nutrition knowledge is a strong predictor of children’s nutrition knowledge, meaning that when parents possess better nutrition knowledge, children are more likely to differentiate between healthy and unhealthy foods [55]. However, most studies at the household level were shorter in duration and of cross-sectional, quasi-experimental, or mixed-method designs, highlighting a lack of longer-term and longitudinal research in families.

Although the majority of studies at the household level focused on parental demographics and characteristics such as parents’ age, education, nutrition knowledge, involvement in meal prep and cooking activities, there is a paucity of studies examining the influence of food access on child food literacy among families with lower SES, which may have contributed to the mixed findings in our scoping review. It is critical to address the issue of lack of access to food at the household level as it may be associated with low self-efficacy to choose healthy foods and food literacy in children [45, 56]. This is unsurprising, as children who usually do not have a choice would likely have difficulty making one when it is available to them. Food insecure children also had lower levels of food literacy compared to those in food secure households, mostly attributed to a lack of household income, poor food access, and the increased cost of healthy foods [38, 45, 46, 55–57, 59]. Therefore, addressing the lack of food access may be an important first step for programs and policies focused on improving food literacy and food-related behaviors in children.

Although child food literacy development may begin at home with parents, schools are often described as “promising spaces” where children can increase their knowledge of healthy eating, cooking skills, food safety, healthier dietary behaviors, and develop a positive relationship with food and the food system by sustaining our planetary health [178, 179]. Evidence from a systematic review suggested that interventions promoting food literacy in schools were associated with improved food literacy in children [179]. A common way to teach children about food literacy in schools was through school gardens, hands-on meal preparation and cooking classes, nutrition education, and modification of the broader school environment. Through planting, growing, harvesting, cooking, and eating fresh foods from school gardens, students improved their food literacy by learning about where food comes from as well as the story behind its transformation [180]. Children involved in gardening and cooking programs at schools were more motivated to develop the cooking skills and knowledge to help them navigate through the intricacies of food systems and help them choose healthier foods. Most school food literacy intervention programs such as school gardens, cooking classes, food tastings, farm to school also reported an improvement in outcomes such as self-efficacy, behavior intent and attitudes [151]. Moreover, exposure to interventions promoting sustainable foods and food systems at schools is said to reinforce students’ knowledge about food systems and attitudes towards eating healthy and sustainable foods such as fruit and vegetables [117], thereby contributing to improved food literacy. Other than interventions targeting the individual level in schools, modifying the school environment such as a federal school lunch program to increase accessibility of whole grains and vegetables was another means to improve child food literacy [11]. An important aspect of the school programs is the involvement of children’s families through newsletters, take-home recipes, and parental engagement in food-related activities since school-aged children still depend on their parents and eat at home. Unfortunately, although school programs had positive effects on children’s food literacy, they faced multiple barriers such as a lack of funding, infrastructure, volunteers, and trained teachers [181].

While various interventions (*n* = 84; 60.4%) in this scoping review have studied the influence of school programs and environment on child food literacy, only one study assessed the influence of child care environment on children’s food literacy. Studying the child care environment is of critical importance because many children throughout the world spend significant time in child care [182, 183]. The child care environment interacts at the individual, interpersonal, and organization levels, and not fully examining its impact on child food literacy remains a major gap in the literature. Furthermore, in our search, we did not find any studies examining the influence of policies on child food literacy at a wider level outside the home and school environment. Globally, large-scale public policies such as poverty reduction and tobacco taxes have been designed to target the wider level.

Although various countries have made considerable efforts to develop programs and policies to improve food literacy to encourage healthy eating in children while improving their food environment [184], there is still a lack of studies examining the influence of policies on child food literacy as measuring its impact on the individual is challenging. Moreover, resistance from the food industry and limited cooperation from governmental agencies to translate “policy into action” may have also contributed to the lack of research on either policy or industry influence on child food literacy [185]. Although food literacy is often seen as an individual attribute, it is essential for individuals or children in this case to navigate levels through complex food systems to be able to choose a healthier and more sustainable diet since the environments that sustain these choices are forged beyond the individual level. This makes it more essential for policymakers to develop policies with food systems in mind [186]. An example is the National School Food Policy in Canada that recently received funding of $1 billion to create a National School Food Program that is expected to provide healthy and nutritious meals to children so they can succeed in schools, improve their food literacy, and promote practices that are environmentally sustainable [187]. Thus, there is an opportunity in the future to study whether this policy and program will indeed affect children’s food literacy, including their ability to navigate and influence complex food systems.

#### Strengths and limitations

This scoping review has summarized evidence from the literature surrounding socio-ecological factors influencing child food literacy by following guidelines from Arksey and O’Malley [23] as well as both Levac et al. [25] and the JBI [26]. By including a broad assessment of potential factors influencing child food literacy from multiple contexts, this review provides us with a comprehensive understanding of key influences on child food literacy. Another strength of the scoping review is that a comprehensive and systematic search was conducted in 3 different databases without any restrictions on study designs, year of publication, and duration. However, the scoping review had a few limitations. First, the search was limited to peer-reviewed publications and did not include grey literature, which may have contributed to the lack of assessment of the policy level. The included studies were also restricted to those in the English language, which further limited our search and may have underestimated the quantity of studies obtained. Moreover, the diversity in the study designs and reported influences suggest that research surrounding child food literacy is still at an early stage, characterized by limited clarity in its definition and a lack of validated tools. In addition, since many dimensions of food literacy are interrelated, the reported associations across our included studies may reflect overlapping measures of the same construct rather than independent relationships. Studies with study designs other than randomized controlled trials were also subject to confounding, questioning the robustness and generalizability of the data. Variability in whether and how the studies adjusted for potential confounders further limits the interpretation of study results and comparability of reported associations across included studies.

## Conclusion

Although there has been a plethora of studies in the past decade on food literacy in children, this scoping review emphasizes the need for future research to focus on longitudinal studies among families. Our findings also highlight the need to develop and validate a more comprehensive and theoretically grounded tool to measure food literacy in children. Findings in this scoping review further demonstrate that most research on child food literacy occurs at the individual, organizational, and household levels and highlight that fewer studies have examined factors at community and policy levels. Since food literacy is a multidimensional concept [21], the focus should not only be on the individual’s knowledge of food and nutrition, but also on the awareness of food systems and the influences of these systems on behavior, recognizing the importance of the food systems dimension of food literacy. Food literacy development and education do not only happen in the home environment but also at the community level. Therefore, to successfully implement programs and policies around child food literacy, a systems-based approach targeting all levels of the socio-ecological model is warranted, and essential to empower individuals not only for individual choices but also to be actors in shaping their food environment and systems where possible [172]. There is still a greater need to converge toward hands-on approaches to garner children’s interest in healthy and sustainable diets for a more comprehensive and holistic approach to food literacy. Based on the evidence reviewed, the representation of our results across the levels of the socio-ecological model may be a starting point for interdisciplinary mapping to inform policy research.

## Supplementary Information

Supplementary Material 1: Table S1. Search Strategies for Medline, Web of Science and CINAHL. Table S2. Characteristics of the included studies. Table S3. Definitions, measurement tools, and socio-ecological influences on child food literacy in included studies.

Supplementary Material 2: PRISMA checklist. 

## Data Availability

The data underlying this scoping review are available within this article, complete with references. The review protocol was also registered with *Open Science Framework* and can be accessed at https://osf.io/9p7n5.
