# Supplementary material for: Mapping the socio-ecological influences on child food literacy: a systematic scoping review
Source: Syst Rev. 2026 Mar 24;15:150. doi: 10.1186/s13643-026-03159-0 (PMC13134327; doi:10.1186/s13643-026-03159-0)
Supplement: Supplementary file 1 — Supplementary Material 1: Table S1. Search Strategies for Medline, Web of Science and CINAHL. Table S2. Characteristics of the included studies. Table S3. Definitions, measurement tools, and socio-ecological influences on child food literacy in included studies. [file 13643_2026_3159_MOESM1_ESM.docx]

**Supplementary Material**

**Supplementary Table S1. Search Strategies for Medline, Web of Science and CINAHL**

| **#** | **Search terms – Medline** |
| --- | --- |
| 1 | exp Food/ |
| 2 | exp Diet/ |
| 3 | exp Feeding Behavior/ |
| 4 | exp Eating/ |
| 5 | 1 or 2 or 3 or 4 |
| 6 | exp Literacy/ |
| 7 | 5 and 6 |
| 8 | ((food* or nutrition*) adj3 (litera* or skill*)).mp. |
| 9 | 7 or 8 |
| 10 | child*.mp |
| 11 | preschool*.mp. |
| 12 | 10 or 11 |
| 13 | 9 and 12 |
| 14 | limit 13 to English language |
| 15 | limit 14 to animals |
| 16 | 14 not 15 |
| # | **Search terms – Web of Science** |
| 1 | TS=(((food* or nutrition* or diet* or eating) NEAR/3 (skill* or litera*))) |
| 2 | TS=(child* or preschool*) |
| 3 | ((TS=((food* or nutrition* or diet*) NEAR/3 (skill* or litera*)))) AND TS=(child* or preschool*) |
| 4 | Refined by: Languages: English |
| # | **Search terms – CINAHL** |
| S1 | (MH "Food+") |
| S2 | (MH "Diet+") |
| S3 | (MH "Eating") |
| S4 | (MH "Eating Behavior+") |
| S5 | S1 OR S2 OR S3 OR S4 |
| S6 | (MH "Literacy") |
| S7 | S5 AND S6 |
| S8 | (("food*" OR "nutrition*") N3 ("litera*" OR skill*)) |
| S9 | S7 OR S8 |
| S10 | child* |
| S11 | preschool* |
| S12 | S10 OR S11 |
| S13 | S9 AND S12  Narrow by Language: - English |

**Supplementary Table S2. Characteristics of the included studies**

| **Author, year [reference]** | **Country of origin** | **Study design** | **Setting** | **Target Population (age), (sample size)** | **Study aims/purpose** | **Intervention** | **Funding** |  |  |  |  |  |  |  |  |  |  |  |  |  |
| --- | --- | --- | --- | --- | --- | --- | --- | --- | --- | --- | --- | --- | --- | --- | --- | --- | --- | --- | --- | --- |
| Adedokun 2020 [1] | USA | Pre-post test | Community kitchens | Grade 3-6, (N=495) | To evaluate the impact of a nutrition education program, on participants' nutrition knowledge, food preparation skills, cooking self-efficacy, and intention to increase fruit and vegetable intake. | Super Star Chef - a 3-day program with ~12hr of instruction on nutrition education and cooking. | Agency |  |  |  |  |  |  |  |  |  |  |  |  |  |
| Ahmadpour 2023 [2] | Iran | Mixed-methods | School | 10-12 years, (N=300) | To design and evaluate an intervention to improve food literacy in Iranian children using the intervention mapping. | Nutrition education and environmental interventions for 16 weeks (a total of 16 sessions). Environmental interventions included school environment intervention that targeted socio-cultural, physical, economic, and political environment, by: (1) modifying the school’s Socio-Cultural environment through implementing simple and healthy food competitions, food and nutrition festivals, healthy nutrition painting contests among children; (2) improving the school’s physical environment through distributing healthy snacks and hygienic foodstuffs at the school canteen; (3) modifying the school’s economic environment by distributing healthy school meals at an affordable price or partially free of charge in the canteen; and (4) improving the school’s political environment by initiating a School Food and Nutrition Committee. | Agency |  |  |  |  |  |  |  |  |  |  |  |  |  |
| Ali 2021 [3] | Spain | Pre-post test | School | 6-11 years, (N=40) | To design a culinary education program and assess its impact on knowledge, phobias, culinary skills and diet quality in school children. | A program with the aim of promoting healthy eating behaviors using cooking as an educational tool. It lasted for one school year with 30 classes (1 hr/class). | Not reported |  | 23.7410072 | 41.0071942 | 26.618705 | 100 |  |  |  |  |  |  |  |  |
| Amin 2018 [4] | USA | Qualitative (focus groups with semi-structured questions) | School | 9-12 years, (N=31) | To broaden the understanding of food literacy with the aim to develop educational and measurement tools. | Focus groups questions informed by food literacy were developed and child food literacy domains were discussed. | Agency |  |  |  |  |  |  |  |  |  |  |  |  |  |
| Anderson 2005 [5] | UK | Pre-post test | School | 8 years (mean), (N=135) | To assess the impact of a school-based nutrition education intervention aimed at increasing the consumption of fruit and vegetables. | A 9-month intervention programme increased the provision of fruit and vegetables in schools and provided point-of-purchase marketing materials, newsletters for children and parents, and teacher information. Curriculum materials included food preparation and tasting, promoted through hands-on activities, written work, videos, self-monitoring materials and story book. | Agency |  |  |  |  |  |  |  |  |  |  |  |  |  |
| Auld 1999 [6] | USA | Quasi-experimental, pre-post test | School | 3-4th grade, (N=543) | To examine the overall consumption of whole grains, fruit and vegetables, knowledge on fruit and vegetables and Food Guide Pyramid and 5 A Day, and food preparation skills. | The intervention consisted of 24 weekly hands-on activities taught by a resource teacher and 6 parent-taught lunchroom "mini-lessons." | Industry and Agency |  |  |  |  |  |  |  |  |  |  |  |  |  |
| Austin 2018 [7] | USA | Pre-post test | Community | 11 years (mean), (N=100 parent-child dyads) | To test whether a family-based media literacy education increases critical thinking about food marketing and decreases children's susceptibility to it. | 6 weekly family-based lessons on marketing environment, marketing strategies to make food more appealing, and how to make healthy food choices. | Agency |  |  |  |  |  |  |  |  |  |  |  |  |  |
| Austin 2020 [8] | USA | Pre-post test | Community | 11.5 years (mean), (N=189 parent-child dyads) | To test the effectiveness of a family-centered, media literacy-oriented intervention to empower parents and children to analyze critically food media messages and improve nutrition skills and behaviours. | Food Mania! - a family-based lessons on understanding nutrition labels and unrealistic food advertising for ~ 2 weeks. | Agency |  |  |  |  |  |  |  |  |  |  |  |  |  |
| Bai 2018 [9] | South Korea | Quasi-experimental, pre-post test | School | 2nd Grade, (N=71) | To explore determinants of vegetable consumption behavior and intention, and to examine the impact of Veggiecation on vegetable consumption behavior, intention and personal factors in children. | Veggiecation - a 4-week program consisting of a 40 min/week classroom lecture and hands-on cooking activities focused on introducing new vegetables. | Not reported |  |  |  |  |  |  |  |  |  |  |  |  |  |
| Banos 2013 [10] | Spain | Cluster randomized controlled trial | School | 11.2 years (mean), (N=228) | To study the efficacy and acceptability of an online game, ETIOBE Mates designed to improve children’s nutritional knowledge. | ETIOBE Mates - a website that combines the Internet and computer games to impart nutritional and healthy lifestyle knowledge for the prevention and treatment of obesity in children. | Agency |  |  |  |  |  |  |  |  |  |  |  |  |  |
| Barton 2005 [11] | USA | Qualitative (focus groups with semi-structured interviews) | School | 10 years (mean), (N=24) | To understand how urban children in poverty view food and food systems and how they use and transform that knowledge in everyday life. | N/A | Agency |  |  |  |  |  |  |  |  |  |  |  |  |  |
| Beck 2021 [12] | Denmark | Pre-post test | Community-local cooking classes | 10.9 years (mean), children (N=82) | To assess the impact of intergenerational cooking activities on dietary habits, food courage, cooking skills and two-way interaction between children and their grandparents. | The Grandchildren’s Food Workshop- an experimental and intergenerational cooking program for grandchildren and grandparents (4 three-hour workshops between 1 to 2 months). | Agency |  |  |  |  |  |  |  |  |  |  |  |  |  |
| Beckman 2008 [13] | USA | Pre-post test | Community | 11.5 years (mean), (N=66) | To evaluate dietary behaviors, nutrition/gardening knowledge among multiethnic inner-city youth using the expanded Theory of Planned Behavior. | Youth Farm and Market Project (YFMP) - a 10-week garden program with inner city youth where they learn about environmental responsibility, cultural diversity, and the food system through gardening, cooking activities and a passive nutrition education curriculum 3 days/week. | Not reported |  |  |  |  |  |  |  |  |  |  |  |  |  |
| Bell 2018 [14] | USA | Quasi-experimental, pre-post test | School | 9.9 years (mean), (N=180) | To examine the effect of the Virtual Sprouts intervention, an interactive multiplatform mobile gardening game, on dietary intake and psychosocial determinants of dietary behavior in minority youth. | A 3-week program that included three Virtual Sprouts gaming sessions, three in-school lessons, and three in-home activities, using a nutrition- and gardening focused curriculum.The Virtual Sprouts game includes a series of cooking and gardening | Agency |  |  |  |  |  |  |  |  |  |  |  |  |  |
| Binder 2020 [15] | Austria | Longitudinal | School | 7.80 years (mean), (N=719) | To investigate the main effects and interplay of children’s amount of media consumption and their parents’ food-related mediation styles. | N/A | Agency |  |  |  |  |  |  |  |  |  |  |  |  |  |
| Bisset 2008 [16] | Canada | Cross-sectional | School | Grades 5-6, (N=388) | To provide an intermediate impact assessment of the nutrition intervention Petits cuistots – parents en réseaux (Little Cooks – Parental Networks) on: 1) knowledge, attitude, capacity and experience with regard to nutrition, diet and cookery, and 2) parental and/or family participation in school. | The program component “Little Cooks” is a nutrition workshop run by community dieticians with parental support. Each of the eight annual workshops features a food item and nutrition theme with a recipe for a collective food preparation and tasting experience. | Agency |  |  |  |  |  |  |  |  |  |  |  |  |  |
| Blanchet 2020 [17] | Canada | Cross-sectional | School | 10–11 years, (N=5,244) | To examine the relationship between household food insecurity and children’s involvement in family meal choices and food preparation, and to explore gender differences within these associations. | N/A | Agency |  |  |  |  |  |  |  |  |  |  |  |  |  |
| Block 2012 [18] | Australia | Mixed-methods | School | 8-12 years, (N=592) | To assess the impact of the SAKG (Stephanie Alexander Kitchen Garden) program. | SAKG - a hands on 45-minute garden class with a 1.5 hour kitchen class every week at school where children learn how to grow, harvest, prepare, share foods, and form positive food habits | Agency |  |  |  |  |  |  |  |  |  |  |  |  |  |
| Brennan 2021 [19] | Ireland | Cluster randomized controlled trial | School | 6-11 years; 10-11 years, (N=903) | To improve children’s health-related quality of life, wellbeing, food knowledge and dietary habits. | Project Daire - two 6-month interventions (Nourish and Engage) developed for children to improve their food knowledge and interests in food. Nourish - a program aimed to change the whole school food environment and increase exposure to local foods; policy recommendations and equipment to enhance school food presentation in canteens e.g. posters, tablecloth. Engage - an age-appropriate, cross-curricular educational intervention on food, agriculture, food and nutrition-related science. | Agency |  |  |  |  |  |  |  |  |  |  |  |  |  |
| Brown 2020 [20] | Canada | Mixed-methods | School | 9-12 years (N=30) | To describe the design, development, and testing of an evidence-based mHealth nutrition education app, Foodbot Factory. | Foodbot Factory - 5 app user testing sessions were conducted among students where engagement and usability were assessed. | Agency |  |  |  |  |  |  |  |  |  |  |  |  |  |
| Burrows 2015 [21] | Australia | Pre-post test | School | 9 years (mean), (N=51) | To evaluate the impact of Back to Basics (B2B) after-school cooking club on dietary behaviours and fruit and vegetable variety in a population at risk of obesity in low-income neighbourhood with a relatively high indigenous population. | 5 (90-minute) cooking sessions after-school, once every 2 weeks during 1 school term where parents and children sat as a group to taste and discuss meal that children prepared. | Agency |  |  |  |  |  |  |  |  |  |  |  |  |  |
| Caraher 2013 [22] | UK | Quasi-experimental | School | 9-11 years, (N=169) | To assess the impact of a teaching program on food, health, nutrition and cookery. | The program consisted of sessions on healthy eating, experiencing food through the senses, with a focus on taste followed by a cooking session. | Agency and Industry |  |  |  |  |  |  |  |  |  |  |  |  |  |
| Cason 2001 [23] | USA | Pre-post test | Daycare | 4.3 years (mean), (N=6,102) | To enable preschool children to identify nutritious snack foods, identify and name fruit and vegetables, increase willingness to taste them, prepare and consume nutritious foods, and acquire behaviors contributing to healthier food choices and lifestyle. | Curriculum developed around healthy snacking, identification of fruit and vegetables, and the Food Guide Pyramid that consisted of 12 lessons (40 mins/session) every 2 weeks. | Agency |  |  |  |  |  |  |  |  |  |  |  |  |  |
| Castagnoli 2023[24] | Brazil | Pre-post test | School | 7-10 years, (N=410) | To evaluate the effect of interdisciplinary educational interventions on children’s attitudes, knowledge, preferences, and perceptions about different nutrition labels | For the first week, the intervention consisted of nutrition information, labels and the relationship between nutrition information and chronic diseases, as well as a nutrition labeling game. For the second week, there was a review of first week concepts, types and location of nutrition labels and their interpretation with a packaging ranking game. | Agency |  |  |  |  |  |  |  |  |  |  |  |  |  |
| Chen 2014 [25] | USA | Mixed-methods | School | 6.1 years (mean), (N=378) | To evaluate the impact of a pilot intervention promoting ethnic produce through classroom food demonstrations, tastings and home cooking activities in ethnically diverse elementary school children in a low-income neighbourhood. | 1-2 ethnic recipes were taught in class monthly tasting activities for 4 months, followed by children receiving recipe kits to make at home. | Agency |  |  |  |  |  |  |  |  |  |  |  |  |  |
| Chu 2013 [26] | Canada | Cross-sectional | School | Grade 5, (N=3,398) | To examine the association between frequency of home meal preparation, fruit and vegetable preference, and self-efficacy for selecting healthy foods. | N/A | Agency |  |  |  |  |  |  |  |  |  |  |  |  |  |
| Colby 2019 [27] | USA | Randomized controlled trial | Community | 9.4 years (mean), (N=228 parent-child dyads) | To describe the challenges and facilitators to incorporating technology into the cooking program. | iCook 4-H Program- a 12-week long with 6 in-person sessions (2 hr/session) with children and an adult meal preparer (parent/grandparents or another adult) to increase cooking, eating, and playing together, while creating and posting videos online. Participants received an additional 21 months of website activities, monthly newsletters, and quarterly in-person booster sessions | Agency |  |  |  |  |  |  |  |  |  |  |  |  |  |
| Colley 2022 [28] | Canada | Cross-sectional | School | 11.2 years (mean), (N=2,443) | To investigate school children’s food and nutrition knowledge and to identify sociodemographic factors related to children’s food related knowledge. | N/A | Agency |  |  |  |  |  |  |  |  |  |  |  |  |  |
| Condrasky 2010 [29] | USA | Pre-post test | Community | 12 years (mean), (N=99) | To examine the impacts of Cook like a Chef on its participants’ knowledge, confidence and motivation in choosing healthy food choices, and acquiring food preparation skills. | Cook like a Chef - 5 full-days (7 hours/day) long hands-on culinary nutrition program designed to encourage healthy eating behaviors among children by building confidence and motivation in cooking skills, nutrition knowledge while involving a chef/instructor, faculty, and dietetic students. | Agency |  |  |  |  |  |  |  |  |  |  |  |  |  |
| Cunningham-Sabo 2014 [30] | USA | Quasi-experimental, pre-post test | School | 8-12 years, (N=961) | To compare effects of the Cooking With Kids (CWK) cooking and tasting curriculum (CWK-CT) with a less-intense, tasting-only curriculum (CWK-T) and to compare fourth graders’ cooking self-efficacy, cooking attitudes, and fruit and vegetable preferences. | Cooking With Kids (CWK) - a school-based experiential nutrition education program of 16 hours of cooking and tasting lessons that engages elementary school children in hands on learning during the school year. | Agency |  |  |  |  |  |  |  |  |  |  |  |  |  |
| Dai 2022 [31] | Taiwan | Quasi-experimental, pre-post test | School | 9-10 years, (N=49) | To determine a change in knowledge and children's perceptions after participating in the intervention. | Traffic Light Diet - a 4- minute class-based nutrition intervention on healthy eating program/week for 8 weeks in which children learn about the caloric values of foods as well as categorizing them. | None |  |  |  |  |  |  |  |  |  |  |  |  |  |
| Dallant 2024 [32] | France | Multi-phase study design with study tool development and validation | School | 9.6 years, (N=1,187) | To develop a questionnaire and to evaluate its measurement properties among French schoolchildren aged 8–11 years. | N/A | Agency |  |  |  |  |  |  |  |  |  |  |  |  |  |
| Davis 2016 [33] | USA | Exploratory cluster randomized controlled trial (RCT) | School | 9.3 years (mean), (N=304) | To evaluate the effect of an exploratory 12-week nutrition, cooking and gardening RCT (“LA Sprouts”) on preference for fruit and vegetables (FV); willingness to try FV; identification of FV; self-efficacy to garden/eat/cook FV; motivation to garden/eat/cook FV; attitudes towards FV; nutrition and gardening knowledge; and home gardening habits. | LA Sprouts - a 90-minute session 1x/week for 12 weeks on cooking, nutrition, and gardening in an outdoor community garden. | Agency |  |  |  |  |  |  |  |  |  |  |  |  |  |
| Dawson-McClure 2014 [34] | USA | Pre-post test | School | 4.63 years (mean), (N=91) | To evaluate the impact of ParentCorps program among children in low-income communities. | ParentCorps - a 5-month long intervention (14 sessions- 2 hour each) developed to promote effective parenting in disadvantaged neighbourhoods to prevent obesity in children. | Not reported |  |  |  |  |  |  |  |  |  |  |  |  |  |
| Depboylu 2023 [35] | Turkey | Cross-sectional | School | 11.3 years (mean), (N=1,074) | To determine the nutrition literacy status of children and its association with adherence to the Mediterranean diet. | N/A | None |  |  |  |  |  |  |  |  |  |  |  |  |  |
| Dixon 2014 [36] | USA | Pre-post test | Community | 12 years (mean), (N=53) | To instruct children on menu planning and healthy eating, and to examine whether teaching children on meal planning would increase fruit and vegetable intake and help them learn how to plan healthier meals. | Cook like Chef - a cooking program where children attend the camp for 5 days for 5 hours/day where they learn food preparation, cooking techniques, nutrition lessons, and menu planning lessons. | Not reported |  |  |  |  |  |  |  |  |  |  |  |  |  |
| Doustmohammadian 2019 [37] | Iran | Cross-sectional | School | 10–12 years, (N=803) | To assess food and nutrition literacy, and its predictors among children. | N/A | Agency |  |  |  |  |  |  |  |  |  |  |  |  |  |
| Doustmohammadian 2022 [38] | Iran | Cross-sectional | School | 10–12 years, (N=803) | To investigate the association between food and nutrition literacy components and eating behaviors, academic achievement, and weight status in school children. | N/A | Agency |  |  |  |  |  |  |  |  |  |  |  |  |  |
| Elsborg 2022 [39] | Denmark | Cluster-based randomized controlled trial | School | 12 years (mean), (N=640) | To evaluate the efficacy of an intervention on food preparation and eating healthy in children. | FOODcamp - a 1-week camp camp so children can learn food preparation skills and to encourage a healthier lifestyle. | Agency |  |  |  |  |  |  |  |  |  |  |  |  |  |
| Ensaff 2017 [40] | UK | Quasi-experimental | School | 7-9 years, (N=663) | To assess a school-based intervention. | Jamie Oliver's Kitchen Garden Project - an intervention that consists of kitchen classroom sessions (90 minutes) where children prepare, cook, and eat food together every 2 weeks during the school year. | Agency |  |  |  |  |  |  |  |  |  |  |  |  |  |
| Espinosa-Curiel 2020 [41] | Mexico | Pre-post test | School | 9 years (mean), (N=60) | To design and test the serious video game FoodRateMaster targeting children | The game includes nutritional information and behavior change techniques to help children improve their knowledge of healthy and unhealthy foods, increase their intake of healthy food, and reduce their intake of ultra processed food. In addition, FoodRateMaster was designed as an active game to promote physical activity. | Agency |  |  |  |  |  |  |  |  |  |  |  |  |  |
| Evans 2012 [42] | USA | Pre-post test | School | Grades 6-7, (N=214) | To measure the effects of different levels of exposure to Sprouting Healthy Kids (SHK) on children's fruit and vegetable consumption and on fruit and vegetables-related psychosocial factors in low-income and racially diverse students. | SHK - a 5-month multicomponent intervention classes on healthy food, soil preparation, organic gardening, local food systems, food production and security. Locally grown vegetables incorporated in school lunch menus at least 1x/week. Posters, table tents, and other materials placed in the cafeteria to identify foods as locally grown. Local farmer visits to schools and farm field trips to increase children's knowledge and motivation to try local foods. | Agency |  |  |  |  |  |  |  |  |  |  |  |  |  |
| Eves 2010 [43] | UK | Mixed-methods | School | 5-7 years, (N=732) | To determine knowledge of food hygiene in young children, and facilitators and barriers to application of knowledge. | Age-appropriate activities on food hygiene/storage/were designed, with schoolteachers, to assess pupils’ (5–7 years) knowledge and hygiene-related practices in relation to expected learning outcomes. | Agency |  |  |  |  |  |  |  |  |  |  |  |  |  |
| Frerichs 2016 [44] | USA | Qualitative (focus groups with semi-structured interviews) | School | Grades 3-7, (N=38) | To identify patterns and themes that stemmed from children's discussions of descriptions of food and healthy eating. | N/A | Agency |  |  |  |  |  |  |  |  |  |  |  |  |  |
| Friel 1999 [45] | Ireland | Quasi-experimental | School | 8-10 years, (N=821) | To assess the impact and suitability of a pilot dietary educational program for students. | The education programme comprised 20 -30 min sessions over 10 weeks on eating healthy. | Agency |  |  |  |  |  |  |  |  |  |  |  |  |  |
| Froome 2020 [46] | Canada | Single-blinded, parallel, randomized controlled pilot study | Community (summer camps) | 9 years (mean), (N=73) | To determine if Foodbot Factory effectively improves children’s knowledge of Canada’s Food Guide. | The Foodbot Factory serious game mobile application was developed to support school children in learning about Canada’s Food Guide. Children were randomized to play Foodbot Factory or a control app called “My Salad Shop Bar” for 10–15 min each day over a five day period. | Agency |  |  |  |  |  |  |  |  |  |  |  |  |  |
| Fulkerson 2010 [47] | USA | Randomized controlled trial | Community (community center or church with kitchens) | 8-10 years, (N=44 parent-child dyads) | To develop, implement, and test the feasibility and acceptability of the HOME program. | The HOME program consists of 5 sessions (90 mins/session) of learning meal planning and cooking skills, eating healthy, increasing family meal frequency and creating healthful environment (~3 months). | Agency |  |  |  |  |  |  |  |  |  |  |  |  |  |
| Gan 2019 [48] | Philippines | Cluster randomized controlled trial | School | 8 years (mean), (N=360) | To develop and validate the Nutrition Knowledge Questionnaire and the Healthy Foodie nutrition game application.  To determine the effectiveness of Healthy Foodie nutrition game application. | The "Healthy Foodie" game -a 25-40 min game was designed to educate children about the three food groups (Go, Grow, Glow), the Filipino food plate, traffic light food groups (green, yellow, red), and the food pyramid. ​ | Not reported |  |  |  |  |  |  |  |  |  |  |  |  |  |
| Gao 2022 [49] | China | Cross-sectional | School | 10-12 years, (N=11,384) | To assess the effect of peer influence on dietary and nutrition knowledge. | N/A | Agency |  |  |  |  |  |  |  |  |  |  |  |  |  |
| Gatto 2012 [50] | USA | Quasi-experimental | Community garden | 9.8 years, (mean), (N=104) | To assess the influence of a garden-based intervention, LA Sprouts on behaviour associated with dietary intake and psychosocial factors in overweight or obese children from low socio-economic status. | LA Sprouts - a 90-minute session 1x/week for 12 weeks on cooking, nutrition, and gardening in an outdoor community garden. | Agency |  |  |  |  |  |  |  |  |  |  |  |  |  |
| Gunther 2019 [51] | USA | Quasi-experimental | Community center | 6.9 years, (mean), (N=126) | To assess the impact of a multi-component family meals intervention study, Simple Suppers. | Simple Suppers - a program included 10 (90-min) lessons delivered weekly focusing on planning healthy family meals on a budget, cooking and eating healthy. | Agency |  |  |  |  |  |  |  |  |  |  |  |  |  |
| Ha 2020 [52] | USA | Randomized controlled trial | Home and laboratory | Children, 10.5 years (mean), (N=36) | To examine if food advertising literacy training impacts children’s food decision-making. | 4 sessions of food advertising literacy training with 12 narratives embedded in the 12 training videos for 1 week. | Agency |  |  |  |  |  |  |  |  |  |  |  |  |  |
| Harley 2018 [53] | USA | Pre-post test | School | Children, 12 years (mean), (N=248) | To examine the effectiveness of Youth Chef Academy. | Youth Chef Academy - 6 sessions (2 hr each) classroom-based, culinary and nutrition literacy curriculum promoting intake of wholefood and plant-based diet by helping children explore the connections between foods, food systems and health. | Agency |  |  |  |  |  |  |  |  |  |  |  |  |  |
| Heerman 2021 [54] | USA | Pre-post test | Community center | Children, 8.8 years (mean), (N=369). | To scale-out an experiential teaching kitchen in Parks and Recreating centres after-school programming in a large urban setting among low-income children. | Teaching kitchen sessions on nutrition education, meal prep and cooking skills - each 30 mins long and to be delivered 2x/month during the spring semester. | Agency |  |  |  |  |  |  |  |  |  |  |  |  |  |
| Hermans 2018 [55] | Netherlands | Pre-post test | School | 11.4 years, (mean), (N=108) | To test the short-term effectiveness of the Alien Health Game, a videogame designed to teach elementary school children about nutrition and healthy food choices. | Alien Health - the experimental condition where a game developed to educate children in healthy food choices and the main function of the important macronutrients. Super Shopper - the active control condition where children were asked to select specific groceries (e.g., fruits or vegetables), after which they receive information on the nutritional values of these products. | Agency |  |  |  |  |  |  |  |  |  |  |  |  |  |
| Hojer 2021 [56] | Denmark | Quasi-experimental | School | Children, 12 years (mean), (N=383) | To investigate the effect of a 5-week sensory-based experiential theme course with fish on children’s food literacy and acceptance of fish. | A 5-week sensory-based experiential theme course with fish (10 lectures of 45 min). | Agency |  |  |  |  |  |  |  |  |  |  |  |  |  |
| Hollywood 2022 [57] | UK | Pre-post test | Home | Children, 10.5 years (mean), (N=210) | To assess the effectiveness of a virtual theory-based cooking intervention (Cook Like A Boss Online). | Cook Like A Boss Online - 5 daily videos for a week of cooking intervention to introduce children to a range of food and skills. | Agency |  |  |  |  |  |  |  |  |  |  |  |  |  |
| Hyland 2006 [58] | UK | Qualitative (focus groups with semi-structured interviews) | School | Children, 11-12 years, (N=28) | To investigate the effectiveness of teaching food preparation skills to low-income children. | Food Club - a 2 hr after school program designed to promote food preparation skills and heathier food options in low-income children that lasted for 20 weeks. | Agency |  |  |  |  |  |  |  |  |  |  |  |  |  |
| Jacob 2019 [59] | Canada | Randomized controlled trial | Community | Children, 8-12 years, (N=101) | To measure the influence of the Chefs in Action program on cooking skills, nutrition knowledge, and attitudes towards healthy eating in children. | Chefs in Action - an intervention with 3 cooking workshops, where each workshop was held for 3 weeks. | Agency |  |  |  |  |  |  |  |  |  |  |  |  |  |
| Jarpe-Ratner 2016 [60] | USA | Quasi-experimental | School | Children, 10.5 years (mean), (N=271) | To evaluate the effect of a community-based, experiential cooking and nutrition education program on consumption of fruits and vegetables and associated intermediate outcomes in students from low-income families. | A 10-week (2 h/week) chef-instructor-led program held in cafeteria kitchens after school. | Industry |  |  |  |  |  |  |  |  |  |  |  |  |  |
| Johnson-Jennings 2020 [61] | USA | Mixed-methods | Community (afterschool program within the housing centre) | 5-12 years, (N=7) | To propose an Indigenous-centered approach to childhood obesity including contextualizing risks, traditional ecological knowledge, and gardening as an intervention in children and their families facing food insecurity and homelessness. | An indigenous afterschool program on indigenous obesity prevention with parents' involvement emphasizing on ecological knowledge, cooking, ancestral gardening and traditional foods. Children met 3x/week during the school year and 4x/week during the summer to garden. | Agency |  |  |  |  |  |  |  |  |  |  |  |  |  |
| Johnston 2018 [62] | USA | Pre-post test | School | Grades 3-4, (N=83) | To describe the results of The Food Doctors (TFD) in elementary school students. | The Food Doctors (TFD) - a three 1.5-hr hands-on, interactive, in-class nutrition education pilot programme for elementary school students, where media, humour, music and dance were included. | None |  |  |  |  |  |  |  |  |  |  |  |  |  |
| Jung 2019 [63] | USA | Pre-post test | School | Kindergarten-Grade 5, (N=1,472) | To examine the effectiveness of a school-based healthy eating intervention program for improving healthy eating knowledge and healthy food choice behavior among children. | Healthy Highway Program teaches and reinforces healthy food literacy education and cafeteria activities on healthy foods and basic nutrition with a road safety analogy. | Not reported |  |  |  |  |  |  |  |  |  |  |  |  |  |
| Kanellopoulou 2021 [64] | Greece | Cross-sectional | School | 11.2 years (mean), (N=1,150) | To examine whether the status of parental health relates to their children’s nutrition literacy level. | N/A | Not reported |  |  |  |  |  |  |  |  |  |  |  |  |  |
| Karpouzis 2024 [65] | Australia | Cluster non-randomized controlled trial | School | 11.1 years (mean), (N=809) | To conduct an impact and process evaluation of Food Education and Sustainability Training (FEAST), to evaluate its effect on children’s fruit and vegetable intakes, F&V variety consumed, nutrition knowledge, food preparation/cooking skills, self efficacy and behaviours, food waste knowledge and behaviours, and food production knowledge. | FEAST- a 10-week curriculum-aligned program, designed to educate children about healthy eating, food waste, and sustainability, while teaching cooking skills. | Agency |  |  |  |  |  |  |  |  |  |  |  |  |  |
| Katz 2011 [66] | USA | Cluster randomized controlled trial | School | 7-9 years, (N=1,180) | To evaluate the effects of a nutrition education program designed to teach children and their parents, and to distinguish between more healthful and less healthful choices. | Nutrition Detectives - 4 mini lessons (20 mins/lesson) on selection of healthy foods and nutrition labelling. | Not reported |  |  |  |  |  |  |  |  |  |  |  |  |  |
| Katz 2014 [67] | USA | Pre-post test | School | 5th Grade, (N=212) | To investigate the impact of a condensed version of a workshop on children's nutrition knowledge and food label literacy. | A 45-minute nutrition lesson on eating healthy and nutrition labelling. | Agency |  |  |  |  |  |  |  |  |  |  |  |  |  |
| Kelly 2022 [68] | Australia | Mixed-methods | School | 4-12 years, (N= Not reported) | To examine food literacy promotion in the classroom across primary schools participating in HealthLit4Kids. | HealthLit4Kids - a 1-year long program designed to build the health literacy of school students. | Agency |  |  |  |  |  |  |  |  |  |  |  |  |  |
| Khorramrouz 2020 [69] | Iran | Cross-sectional | School | 9-12 years, (N=315) | To examine the relationship between household food insecurity and food literacy in children. | N/A | Agency |  |  |  |  |  |  |  |  |  |  |  |  |  |
| Knapp 2019 [70] | USA | Qualitative (focus groups with semi-structured interviews) | School | 12 years (median), (N=27) | To examine student, parent, teacher perceptions of ESYNOLA, a school-based kitchen garden program. | A qualitative study design was used to examine the perceptions of and values associated with participation in school-based kitchen garden programs, ESYNOLA. | Agency |  |  |  |  |  |  |  |  |  |  |  |  |  |
| Koch 2006 [71] | USA | Pre-post test | Community | Grades 2-5, (N=56) | To evaluate the effect of a garden program, Health and Nutrition from the Garden on the nutritional knowledge, attitudes, and behaviors of children. | Health and Nutrition from the Garden - a 16-week program with a focus on gardens, basic gardening, growing techniques, healthy eating, healthy snack and food safety. | Not reported |  |  |  |  |  |  |  |  |  |  |  |  |  |
| Kocyigit 2025 [72] | Turkey | Pre-post test | School | 5-6 years, (N=45) | To evaluate the effectiveness of nutrition education in improving recognition and differentiation of food groups and healthy and unhealthy food choices in preschool children. | A 4-week, 30 min-nutrition education intervention that aimed to improve preschool children's knowledge of food groups and their ability to differentiate between healthy and unhealthy food choices. | Agency |  |  |  |  |  |  |  |  |  |  |  |  |  |
| Labbé 2023 [73] | Canada | Quasi-experimental | School | 10.6 years (mean), (N=149) | To assess the impact of a school-based culinary programme on children's food literacy and vegetable, fruit, and breakfast consumption. | A 6-week, 45-min intervention that consisted of culinary workshops on healthy eating, food and nutrition, how to read and follow recipes, preparing and cooking foods using various culinary techniques, and applying food safety practices. | Agency |  |  |  |  |  |  |  |  |  |  |  |  |  |
| Lakshman 2010 [74] | UK | Cluster randomized controlled trial | School | 10.5 years (mean), (N=1,133) | To assess the effectiveness and acceptability of an educational intervention to increase nutrition knowledge in school children. | The program consisted of nutrition education activities related to healthy eating and lasted for 9 weeks during the summer term. | Agency |  |  |  |  |  |  |  |  |  |  |  |  |  |
| Liao 2016 [75] | Taiwan | Pre-post test | School | 10-11 years, (N=140) | To evaluate the impact of children's food advertising literacy program on nutrition knowledge, food advertising literacy, and food purchasing behaviours. | The intervention consisted of 3 groups: Group A received the food advertising literacy program; Group B received a comparable knowledge-based nutrition education program, and the control group did not receive any nutrition education. The 6-week intervention included one 40-min lesson per week. | Agency |  |  |  |  |  |  |  |  |  |  |  |  |  |
| Liquori 1998 [76] | USA | Quasi-experimental, pre-post test | School | Kindergarten - Grade 6, (N=590) | To examine the feasibility and effectiveness of a nutrition education intervention, the Cookshop Program. | Cookshop Program - a program with a school lunch component in the aim to increase whole grains and vegetables in children throughout the school year. It comprised of 10 cooking workshops and 10 food and environment lessons, parents were provided with monthly newsletter on buying, storing, and preparing foods. | Agency |  |  |  |  |  |  |  |  |  |  |  |  |  |
| Liu 2021 [77] | China | Cross-sectional | School | 12 years (mean), (N=4,359) | To identify the dimensions and core components of food and nutrition literacy. | N/A | Agency |  |  |  |  |  |  |  |  |  |  |  |  |  |
| Mack 2020 [78] | Germany | Cluster randomized controlled trial | School | 9.7 years (mean), (N=82) | To evaluate the newly developed game and to evaluate how well children are able to understand and apply the dietary energy density principle (DED-P). | In the intervention group, children played a game about nutrition and healthy lifestyle designed for obesity prevention in children twice over a 2-week period. | Agency |  |  |  |  |  |  |  |  |  |  |  |  |  |
| Maiz 2021 [79] | Spain | Quasi-experimental | School | 8.7 years (mean), (N=196) | To investigate the effect of involving children in their feeding process (choosing a recipe, purchasing the ingredients, and cooking) on their lunch food choice in a school environment. | EgizuSUK! Project - A three 1-hour workshops (1 workshop/wk), different for each group: Hands-on (HO) cooking-related activities, and nutrition education (NE), healthy habits promotion through nutrition education activities. | Agency and Industry |  |  |  |  |  |  |  |  |  |  |  |  |  |
| Morgan 2010 [80] | Australia | Quasi-experimental, pre-post test | School | 11-12 years, (N=111) | To investigate the impact of school garden-enhanced nutrition education on children’s fruit and vegetable consumption, vegetable preferences, fruit and vegetable knowledge and quality of school life. | The program was a 10-week programme (3x1 hr nutrition education lessons in the classroom) delivered by classroom teachers to students in their normal class groups at school. A series of newsletters were provided to parents on the health benefits of fruit and vegetables, to increase family awareness about healthy food choices. | Agency |  |  |  |  |  |  |  |  |  |  |  |  |  |
| Morgan 2014 [81] | USA | Pre-post test | School | 9 years (mean), (N=105) | To educate children about healthy nutrition, promote physical activity, and use avatars as an educational and motivational tool for understanding and developing healthy bodies. | Intervention is a 10-week nutrition education program that incorporated classroom physical activity and exposure to avatars (a form of technology to enhance learning). | Not reported |  |  |  |  |  |  |  |  |  |  |  |  |  |
| Morris 2001 [82] | USA | Pre-post test | School | 1st Grade, (N=97) | To assess the feasibility of garden-based education programs for elementary-school students. | Intervention consists of nutrition lessons &/or a gardening component taught throughout the school year. | Agency |  |  |  |  |  |  |  |  |  |  |  |  |  |
| Morris 2002 [83] | USA | Quasi-experimental | School | 9-10 years, (N=213) | To evaluate the effectiveness of a 1-year nutrition program designed to improve nutrition knowledge in school-aged children. | Intervention consists of 9 nutrition lessons &/or a gardening component for 17 weeks. Newsletters were sent home to students' families (intervention groups) to reinforce concepts taught in class and instigate family discussion. | Agency |  |  |  |  |  |  |  |  |  |  |  |  |  |
| Moss 2013 [84] | USA | Quasi-experimental, pre-post test | School | 3rd Grade, (N=65) | To introduce Coordinated Approach to Child Health (CATCH) and Farm to School program and assess the nutrition knowledge of 3rd grade students. | CATCH - intervention consists of 2 nutrition education classes with physical activity ranging ~30 mins with a farm tour and lasted for 4 weeks. | Agency |  |  |  |  |  |  |  |  |  |  |  |  |  |
| Murad 2021 [85] | USA | Pre-post test | Home | 12 years (mean), (N=17) | To evaluate the effectiveness of a virtual camp focusing on nutrition or cooking classes. | A 5-day long virtual culinary camp focusing on nutrition or cooking lessons. | Agency |  |  |  |  |  |  |  |  |  |  |  |  |  |
| Muzaffar 2014 [86] | USA | Randomized controlled trial | School | Grades 6-8, (N=181) | To improve knowledge, outcome expectations, self-efficacy, and self-reported food intake and skills. | The HOT (Healthy Outcome for Teens) Project - an innovative online educational intervention for middle school children for prevention of diabetes and obesity by balancing food intake with physical activity (5 sessions, 35-40 min long). | Agency |  |  |  |  |  |  |  |  |  |  |  |  |  |
| Nelson 2016 [87] | USA | Pre-post test | School | 3rd Grade, (N=200) | To assess the efficacy of Making Media for a Healthier U (MMHU) for increasing nutrition knowledge in children. | Making Media for a Healthier U (MMHU) - a 9-hour program on food advertising literacy and nutrition education to counteract unhealthy food advertising, encourage healthier food choice, and improve nutrition knowledge. | Not reported |  |  |  |  |  |  |  |  |  |  |  |  |  |
| Ng 2022 [88] | Malaysia | Cluster randomized controlled trial, single-blinded | School | 10-11 years, (N=83) | To evaluate the effectiveness of a culinary nutrition education intervention on children’s home food availability and psychosocial factors related to healthy meal preparation. | 12 weeks of culinary nutrition education with 5 hands-on healthy meal preparation modules and a module with parents on home food availability (conducted every 2 weeks). | Agency |  |  |  |  |  |  |  |  |  |  |  |  |  |
| Nogueira 2022 [89] | Portugal | Community-based participatory research | School | 6-10 years, (N= 1,740 children) | To describe the process of development and implementation of a food literacy curriculum for children. | Health at Table - an intervention focusing on weekly sessions of food literacy and nutrition education. | Agency |  |  |  |  |  |  |  |  |  |  |  |  |  |
| Nozue 2016 [90] | Japan | Cross-sectional | School | 10-11 years, (N=1,207) | To examine children’s involvement in home meal preparation activities and the associations of these activities with food intake and cooking skills. | N/A | Agency |  |  |  |  |  |  |  |  |  |  |  |  |  |
| O'Brien 2006 [91] | USA | Quasi-experimental | School | 9-10 years, (N=38) | To assess the impact of lessons and determine if there is an increase in children’s nutrition knowledge, fruit and vegetable preference, and self-efficacy. | Junior Master Gardener: Health and Nutrition from the Garden Curriculum - the intervention consisted of 8 lessons with a focus on gardening and nutrition lessons (with 30 mins of gardening time) over 10 weeks. | Not reported |  |  |  |  |  |  |  |  |  |  |  |  |  |
| Olan 2019 [92] | USA | Pre-post test | School | 7-8 years, (N=85) | To investigate the implementation of Coordinated Approach to Child Health (CATCH) in 2nd grade students. | CATCH - a 12-week program on nutrition education, healthy food choices, and farm tours implemented for 30 minutes, 2x/week, with a total of 5 lessons. | Not reported |  |  |  |  |  |  |  |  |  |  |  |  |  |
| Overcash 2018 [93] | USA | Pre-post test | Community | 9-12 years (N=89 parent-child dyads) | To evaluate the immediate impact of the vegetable-focused cooking skills program on parent and child psychosocial measures such as child self-efficacy and cooking attitudes in low-income families. | Cooking Matters for families - 6 (2-hour)-weekly sessions for 15 weeks on demonstration, food preparation with parents, and nutrition education. | Agency |  |  |  |  |  |  |  |  |  |  |  |  |  |
| Parmer 2009 [94] | USA | Pre-post test | School | 7.4 years (mean), (N=115) | To examine the effects of a school garden on children’s fruit and vegetable knowledge, preference, and consumption. | Intervention consists of nutrition education (1 hr every other week) &/or a gardening component (1 hr every other week) for 28 weeks. | Not reported |  |  |  |  |  |  |  |  |  |  |  |  |  |
| Perez-Rodrigo 1997 [95] | Spain | Pre-post test | School | 8-12 years (N=150) | To test a school nutrition education program implemented in a deprived urban district. | A 5-week nutrition education program on improving knowledge, skills for choosing healthy foods, resisting peer pressure, and food label reading. | Agency |  |  |  |  |  |  |  |  |  |  |  |  |  |
| Pirouznia 2001 [96] | USA | Cross-sectional | School | 12 years (mean), (N=532) | To examine the correlation of nutrition knowledge and eating behavior in children. | N/A | Not reported |  |  |  |  |  |  |  |  |  |  |  |  |  |
| Policastro 2023 [97] | USA | Pre-post test | School | 10.6 years (mean), (N=30) | To investigate the impact of a culinary literacy curriculum on children’s acceptance of vegetable-added (mushrooms) recipes, self-efficacy to cook, and willingness to try vegetables. | The Healthy Helpers: Culinary Literacy for Kids research study used a 6-week curriculum to teach elementary-aged children CL, including basic cooking skills and concepts. | Agency |  |  |  |  |  |  |  |  |  |  |  |  |  |
| Poston 2005 [98] | USA | Quasi-experimental | Community | 8-11 years (N=29) | To evaluate the effectiveness of a garden-enhanced nutrition curriculum with respect to nutrition knowledge, fruit and vegetable preference, and self-efficacy for gardening and eating fruit and vegetables. | The intervention consisted of 8 lessons with a focus on gardening (20-60 mins) for 8 weeks and 5 nutrition lessons, ranging from (30-60 mins) for 5 weeks. | Agency |  |  |  |  |  |  |  |  |  |  |  |  |  |
| Powell 2018 [99] | USA | Mixed-methods | Community | 7-12 years, (N=27 parent-child dyads) | To examine if a media literacy intervention can increase media literacy knowledge. | The intervention was a 2-hr family-based media literacy educational intervention on the relationship between media literacy and food marketing delivered to parent-child dyads. | Not reported |  |  |  |  |  |  |  |  |  |  |  |  |  |
| Powers 2005 [100] | USA | Pre-post test | School | 7.58 years (mean), (N=1,100) | To determine the effects of a nutrition education program based on the tenets of Social Cognitive Theory (SCT) on dietary behavior and nutrition knowledge among children. | Intervention consists of 6 weekly nutrition classes for 6 weeks. | Agency |  |  |  |  |  |  |  |  |  |  |  |  |  |
| Puma 2013 [101] | USA | Quasi-experimental | School | 5th Grade, (N=308) | To examine the long-term effects of the Integrated Nutrition and Physical Activity Program (INPAP). | INPAP - a 2-year school-based nutrition education program in rural county where students receive free or reduced-price meal plans. | Agency |  |  |  |  |  |  |  |  |  |  |  |  |  |
| Quinn 2003 [102] | USA | Pre-post test | School | 10.7 years (mean), (N=126) | To improve attitudes toward and increase fruit and vegetable consumption of 5th grade students through a modified implementation of the 11-lesson CookshopTM program. | CookshopTM program consists of 11 lessons on different fruit and vegetables, where they are grown, and hands-on cooking with parental involvement. | Agency |  |  |  |  |  |  |  |  |  |  |  |  |  |
| Ratcliffe 2011 [103] | USA | Quasi-experimental, pre–post test | School | 12 years (mean), (N=161) | To investigate the impact of participating in a school garden program in low income, racially and ethnically diverse urban middle students. | Students participated in garden-based learning sessions integrated in their science class - (1 hr a week for 4-month, for a total dose of 13 hrs). | Agency |  |  |  |  |  |  |  |  |  |  |  |  |  |
| Rodriguez 2015 [104] | USA | Qualitative (focus groups with interviews) | School | 9-12 years (N=20) | To identify participants' thoughts, feelings, and perceptions of impacts from their engagement in an extension garden program in low income and racialized communities. | A qualitative design was used to gain insight from a previous extension school-based gardening program. | Not reported |  |  |  |  |  |  |  |  |  |  |  |  |  |
| Rosi 2016a [105] | Italy | Pre-post test | School | 8-11 years (N=8,165) | To assess the impact of early school years of the nutritional intervention within the Giocampus school project. | Giocampus - A 3-month program with different nutrition-theme activities and games (3 hr/class) delivered in Grades 3-5. | Not reported |  |  |  |  |  |  |  |  |  |  |  |  |  |
| Rosi 2016b [106] | Italy | Randomized controlled trial | School | 8-10 years (N=145) | To evaluate if the presence of a humanoid robot could improve the efficacy of a game-based, nutritional education intervention. | Intervention (based on the framework of the Giocampus program)- a game-based nutritional educational lesson on the importance of carbohydrates (1 hr/class) &/or interaction with a humanoid robot that lasted during the school year. | Not reported |  |  |  |  |  |  |  |  |  |  |  |  |  |
| Sahye-Pudaruth 2024 [107] | Canada | Longitudinal | Home-based | 10 years (mean), (N=60) | To examine whether early life involvement in food skills is prospectively associated with cooking skills among children. | The Guelph Family Health Study- a home-based obesity prevention intervention on motivational interviewing and consisted of home visits with a health educator, tailored emails, and mailed incentives, while the control group consisted of general health advice through monthly emails. | Agency |  |  |  |  |  |  |  |  |  |  |  |  |  |
| Sahye-Pudaruth 2025 [108] | Canada | Longitudinal | Home-based | 8.9 years (mean), (N=158) | To examine the longitudinal association between parents’ food skills and children’s cooking skills. | The Guelph Family Health Study- a home-based obesity prevention intervention on motivational interviewing and consisted of home visits with a health educator, tailored emails, and mailed incentives, while the control group consisted of general health advice through monthly emails. | Agency |  |  |  |  |  |  |  |  |  |  |  |  |  |
| Saksvig 2005 [109] | Canada | Pre-post test | School | 10.5 years (mean), (N=122) | To assess the effects of the intervention on knowledge and psychosocial factors related to healthy eating among First Nations children. | Sandy Lake school-based diabetes prevention intervention combines an ecological model and social cognitive theory (SCT) approaches and focus on knowledge and skills development related to healthy eating, physical activity, and diabetes education. This 9-month intervention has family component where parents are informed about healthy eating and physical activity; a peer component that provides opportunities for peers to act as role models; an environment component where the school board /staff developed to school-wide policy to ban high fat and high sugar foods in schools; a low fat/sugar school lunch provided to students at low cost. | Agency and Industry |  |  |  |  |  |  |  |  |  |  |  |  |  |
| Scherr 2017 [110] | USA | Cluster randomized controlled trial | School | 9-10 years, (N=409) | To evaluate the effectiveness of the Shaping Healthy Choices Program (SHCP). | Shaping Healthy Choices Program - a 1 school-year long garden-enhanced education, family, and community partnerships that aims to increase nutrition knowledge; promote availability, consumption and enjoyment of fruit and vegetables; foster positive changes in school environment while creating a community-based support system. | Agency |  |  |  |  |  |  |  |  |  |  |  |  |  |
| Schmidt 2022 [111] | USA | Pre-post test | Community | 9.8 years (mean), (N=180) | To evaluate the effect of a hands-on after-school dietitian-led culinary education program on healthy eating behaviors in a low-income community. | CHEF Bites - a 3-month program with 12 modules (60 mins/module) incorporating nutrition education, culinary skills demonstration and practices, and guided food tasting. | Agency |  |  |  |  |  |  |  |  |  |  |  |  |  |
| Scott 2022 [112] | UK | Qualitative (semi-structured interviews) | Home | 8-10 years (N=42 parent-child dyads) | To explore food-related experiences and changes to behavior of families with children, during the pandemic. | N/A | Not reported |  |  |  |  |  |  |  |  |  |  |  |  |  |
| Shannon 1988 [113] | USA | Pre-post test | School | 3rd-5th Grade, (N=841) | To enhance the nutrition knowledge, attitudes, and self-reported eating behavior of participating children. | The intervention consists of 9-12 weeks of nutrition education on food experiences, nutrition and health. | Agency |  |  |  |  |  |  |  |  |  |  |  |  |  |
| Sharkey 2023 [114] | USA | Mixed-methods | Community | 8-11 years (N=22 children) | To report on the development, implementation, and evaluation of Cooking with the Seasons for Health (CwS4H). | CwS4H- a 6-session nutrition program, with two weekly group sessions during each of three growing seasons. | Not reported |  |  |  |  |  |  |  |  |  |  |  |  |  |
| Smith 2016 [115] | USA | Pre-post test | School | 4th Grade, (N=139) | To determine if a 3-week garden-based pulse nutrition and biology curriculum had a positive impact on knowledge of and preference for dry beans. | Pulse on Health - a STEM-focused school garden curriculum to increase familiarity with pulses through garden lessons and activities for 3 weeks. | Agency |  |  |  |  |  |  |  |  |  |  |  |  |  |
| Smolak 1998 [116] | USA | Pre-post test | School | Kindergarten-5th Grade, (N=222) | To describe the 5th grade curriculum on healthy eating and exercise and evaluate its impact in a rural area. | The curriculum consists of 10 lessons on healthy eating and exercise with a homework assignment supported by parental involvement. | Agency |  |  |  |  |  |  |  |  |  |  |  |  |  |
| Somerset 2009 [117] | Australia | Historical control design | School | 11.5 years (mean), (N=252) | To determine changes in ability to identify specific fruit and vegetables, and attitudes towards fruit and vegetables, associated with the introduction of a school-based food garden. | A 12-month intervention that consists of a school-based food garden and a teacher coordinator for 11 hours/week to facilitate integration of garden activities into the curriculum. | Agency |  |  |  |  |  |  |  |  |  |  |  |  |  |
| Tabacchi 2020 [118] | Italy | Cross-sectional | School | 4.8 years (mean), (N=505) | To investigate the food literacy level in pre-schoolers and to evaluate the effect of potential predictors. | The modules were aimed at assessing children’s knowledge, attitudes and skills on different aspects of food, diet and nutrition. | Agency |  |  |  |  |  |  |  |  |  |  |  |  |  |
| Tabacchi 2021 [119] | Italy | Cross-sectional | School | 4.7 years (mean), (N=79 dyads of children and mothers) | To investigate the extent to which maternal food habits and physical activity level predict food-related aspects in children with lower socio-economic status. | N/A | Agency |  |  |  |  |  |  |  |  |  |  |  |  |  |
| Townsend 2006 [120] | USA | Randomized controlled trial | Community | 9-12 years, (N=5,111) | To examine the effectiveness of a program, Youth Expanded Food and Nutrition Education Program (EFNEP) in low-income families and youth. | EFNEP - an intervention that consists of 7-lesson education experience to enhance knowledge, skills, and food choices via experiential activities that include food tasting, food art, food puzzles, games, and preparation of fruit and vegetables. | Agency |  |  |  |  |  |  |  |  |  |  |  |  |  |
| Treu 2017 [121] | USA | Quasi-experimental | School | 8.7 years (mean), (N=1,487) | To compare a low-dose standard intervention to an enhanced intervention. | Nutrition Detectives - A 90-minute lesson on selection of healthy foods and nutrition labelling. ABC for Fitness (ABC) -30 minutes of physical activity spread over a school day. Enhanced intervention combined Nutrition Detectives, ABC, and reinforcement messages to children and their families. | Agency |  |  |  |  |  |  |  |  |  |  |  |  |  |
| Truman 2019 [122] | Canada | Pre-post test | School | 11 years (mean), (N=71) | To evaluate the influence of media literacy lessons for children by focusing on food marketing. | Media literacy lesson plans for children focusing on critical knowledge around food marketing. | Agency |  |  |  |  |  |  |  |  |  |  |  |  |  |
| Tuuri 2009 [123] | USA | Randomized block design | School | Grades 4-5, (N=560) | To assess the feasibility of the 12-week intervention among children in low-income urban schools. | Smart Bodies - a 12-week program emphasizing consumption of fruit and vegetables delivered through interactive school assemblies, dolls, classroom videos, books, games and lessons, and the immersive learning environment and other interactive activities. | Agency |  |  |  |  |  |  |  |  |  |  |  |  |  |
| Varì 2022 [124] | Italy | Quasi-experimental | School | 12 years (mean), (N=540) | To evaluate the effectiveness of the MaestraNatura program in improving nutrition knowledge in students. | MaestraNatura - a 2-hour intervention focusing on food and nutrition knowledge, food waste, and diet sustainability by supporting both teachers and parents to eventually encourage the family to adopt healthier dietary habits throughout the school year. | Agency |  |  |  |  |  |  |  |  |  |  |  |  |  |
| Varì 2023 [125] | Italy | Quasi-experimental | School | 9-10 years, (N=243) | To evaluate the effectiveness of the MaestraNatura program in improving nutrition knowledge in students. | MaestraNatura - an intervention focusing on food and nutrition knowledge, food waste, and diet sustainability by supporting both teachers and parents to eventually encourage the family to adopt healthier dietary habits throughout the school year. | Agency |  |  |  |  |  |  |  |  |  |  |  |  |  |
| Vaughan 2025 [126] | UK | Cluster randomized controlled trial | School | 6-9 years, (N=631) | To evaluate the impact of a nutrition education intervention, ‘PhunkyFoods’ on food literacy, cooking skills and fruit and vegetable intake in primary school aged children. | Phunky Foods - a school-based nutrition education program aimed at improving food literacy, cooking skills, and fruit and vegetable intake among primary school children | Agency and Industry |  |  |  |  |  |  |  |  |  |  |  |  |  |
| Velardo 2019 [127] | Australia | Qualitative (focus groups and semi-structured interviews) | School | 11-12 years, (N=38) | To understand facilitators and barriers influencing children’s nutrition literacy in a low-income community. | N/A | Not reported |  | Not validated=33 | Partially validated=57 | Validated =37 |  | 139 |  |  |  |  |  |  |  |
| de Vlieger 2021 [128] | Australia | Cluster randomized controlled trial | School | 9-12 years, (N=169) | To investigate the feasibility and acceptability of VitaVillage as a nutrition education tool in primary schools. The | Participants in the intervention group played VitaVillage, a farming-style game designed to improve nutrition for 20 minutes on two occasions over one week, while the control group played mathematics games during the same time. | Not reported |  |  |  |  |  |  |  |  |  |  |  |  |  |
| Weber 2018 [129] | Germany | Controlled, non-randomized | School | 9.6 years (mean), (N=305) | To examine the effects of nutrition lessons among migrant school children. | 3-day practical nutrition lessons comprising of 6 modules on practical handling of foods and kitchen utensils, nutrition education, food preparation, food hygiene, and ability to create a pleasant eating atmosphere during the school year. | Agency |  |  |  |  |  |  |  |  |  |  |  |  |  |
| Wen 2025 [130] | China | Cross-sectional | School | 2-6 years, (N=739) | To develop and validate the Nutrition Literacy Questionnaire for Chinese Pre-school Children (NLQ-PSC). | N/A | Agency |  |  |  |  |  |  |  |  |  |  |  |  |  |
| Whiteley 2015 [131] | Australia | Pre-post test | School | Preschool children (N= ~300) | To increase children's food literacy and attitudes, and knowledge in low socio-economic areas. | Weekly 1–2-hour nutrition lessons (Vegie Fun for Everyone) for 6 weeks to inform children about vegetables and how they are grown. | Not reported |  |  |  |  |  |  |  |  |  |  |  |  |  |
| Wolfe 2018 [132] | USA | Pre-post test | School | Grades 3-5, (N=5,636) | To evaluate the effectiveness of Choose Health: Food, Fun, and Fitness (CHFFF) among children in low-income neighbourhood. | Choose Health: Food, Fun, and Fitness (CHFFF) - a 6-lesson (45 mins/lesson) hands-on, experiential curriculum aimed at 3rd to 6th graders weekly. | Agency |  |  |  |  |  |  |  |  |  |  |  |  |  |
| Woodruff 2013 [133] | Canada | Cross-sectional | School | 11.5 years (mean), (N=145) | To describe family dinner frequency by food preparation frequency (prep), self-efficacy for cooking, and food preparation techniques among children. | N/A | Not reported |  |  |  |  |  |  |  |  |  |  |  |  |  |
| Woodruff 2020 [134] | Canada | Pre-post test | School | 10.2 years (mean), (N=273) | To create and assess the impact of food literacy lesson plans delivered together with a centrally procured school snack program (one fruit or vegetable, 5 days/week for 8 weeks). | Food literacy lesson plans with the use of weekly food delivery menu and grade 5 Ontario curriculum for 8 weeks | Agency |  |  |  |  |  |  |  |  |  |  |  |  |  |
| Wright 2012 [135] | USA | Randomized controlled trial | School | 8-12 years, (N=251) | To measure whether CSHP (Coordinated School Health Programs) with parental, school, and home-based components to promote optimal nutrition will reduce BMI percentiles and z-scores, and improve dietary behaviors in a sample of low-income, school-aged children. | Kids Nutrition and Fitness - a 6-week nutrition, physical activity educational after-school program, and school activities (weekly 90-minute sessions on physical activity, nutrition education) with parental involvement, including creation of an Advisory Committee that made wellness policies and promoted school wellness policies involving school-based physical activity and healthy dietary changes. | Agency |  |  |  |  |  |  |  |  |  |  |  |  |  |
| Xu 2024 [136] | China | Cross-sectional | School | 11.9 years (mean), (N=204) | To examine the relationship between parental food education and children’s food literacy as a means of exploring the mediating roles of the parent–child relationship and learning motivation, as well as the moderating role of the teaching stage. | N/A | Agency |  |  |  |  |  |  |  |  |  |  |  |  |  |
| Yoshii 2021 [137] | Japan | Quasi-experimental, pre-post test | School | Grades 3-4, (N=250) | To assess the impact of the cooking programme on children’s participation in cooking at home and their self-efficacy and attitudes towards cooking. | Students in the intervention school received three 45-minute cooking lessons, including two lessons that focused on peeling apples and one hands-on cooking experience while parents received newsletter. | Not reported |  |  |  |  |  |  |  |  |  |  |  |  |  |
| Zahr 2017 [138] | Canada | Quasi-experimental | School | Grades 4-5 years, (N=100) | To evaluate the influence of Project CHEF, a hands-on cooking and tasting program in public schools, on students’ food preferences, cooking skills, and confidence. | Project CHEF- 4-5 hands-on cooking and tasting program on students’ food preferences, cooking skills, and confidence. | None |  |  |  |  |  |  |  |  |  |  |  |  |  |
| Zarnowiecki 2012 [139] | Australia | Cross-sectional | School | 5.3 years (mean), (N=192) | To investigate whether parents’ nutrition knowledge and attitudes about food predict young children’s knowledge of healthy foods. | N/A | Agency |  |  |  |  |  |  |  |  |  |  |  |  |  |

**Supplementary Table S3. Definitions, measurement tools, and socio-ecological influences on child food literacy in included studies**

| **Author, year [reference]** | **How is food literacy defined or measured?** | **Tool/scale used to measure food literacy and its components** | **Validation Status of food literacy tools** | **Influences on child food literacy and key findings** | **Level of the socio-ecological model** | **What variables were controlled for?** |
| --- | --- | --- | --- | --- | --- | --- |
| Adedokun 2020 [1] | Nutrition knowledge, cooking self-efficacy, and food preparation skills | Survey developed by the research team and adapted from existing measures | No additional validation for the combined tool conducted | Exposure to a program on nutrition education, food preparation, and cooking showed significant improvement in participants' nutrition knowledge, food preparation skills, and cooking self-efficacy. | Individual/Community | None |
| Ahmadpour 2023 [2] | Food choice literacy | Food and nutrition literacy (FNLIT) survey | Validated | Home food environments in increasing students’ access to healthy food and limiting unhealthy food access; students’ involvement in food shopping, preparation, and cooking at home; involving students in home food decisions; home gardening; school’s socio-cultural, physical, economic, and political environment improved food choice literacy in children. | Individual/Household/Organizational | None |
| Ali 2021 [3] | Food knowledge and skills, and culinary skills | Adapted from existing measure - General Nutrition Knowledge Questionnaire validated for use in adults | Not validated | Exposure to a program on healthy eating and cooking skills improved food knowledge and beliefs, and culinary skills. | Individual/Organizational | None |
| Amin 2018 [4] | Food literacy | N/A | N/A | Home food environment and parental involvement in cooking and home gardening influenced food literacy. | Individual/Household | N/A |
| Anderson 2005 [5] | Nutrition knowledge | Pictorial technique to measure fruit and vegetables knowledge developed by the team | Not validated | Exposure to school-based nutrition education intervention and meal prep activities, with changes to school environment increased fruit and vegetable knowledge. | Individual/Household/Organizational | Age and sex |
| Auld 1999 [6] | Nutrition knowledge and self-efficacy to prepare foods | Survey developed by the research team | Not validated | School-based intervention with parental involvement increases nutrition knowledge and self-efficacy to prepare foods. | Individual/Household/Organizational | Pretest scores, gender, ethnicity, and academic status |
| Austin 2018 [7] | Self-efficacy to choose healthy foods and food label literacy | Survey developed by the research team | Internal consistency reported; no additional validation conducted | Exposure to a family-based media literacy curriculum improved parents' media management skills and decreased children's susceptibility to unrealistic food marketing and improved self-efficacy to choose healthy foods. | Individual/Household/Community | Pretest constructs were controlled for (Parents: expectancies for mediation; self-efficacy for dietary changes; negative mediation. Youth: perceived desirability; wishful identification; requests for advertised foods. Parents and youth: use of nutrition facts labels) |
| Austin 2020 [8] | Food label literacy and food advertising literacy | National Collaborative on Childhood Obesity Research (NCCOR) Measures Registry | Validated | Exposure to Food Mania! improved child-initiated discussions about media food messages and food labels. | Individual/Household/Community | SNAP (Supplemental Nutrition Assistance Program), participation, Race/ethnicity, youth age |
| Bai 2018 [9] | Self-efficacy to consume vegetables | Survey developed by the research team | Not validated | Exposure to a program on introducing new vegetables, significantly improved children's self-efficacy to consume vegetables. | Individual/Organizational | Baseline pre-intervention scores |
| Banos 2013 [10] | Nutrition knowledge | Survey adapted from existing validated measures | No additional validation for the combined tool conducted | Exposure to online nutritional games led to improved nutrition knowledge. | Individual/Organizational | Age, sex, and BMI z-score |
| Barton 2005 [11] | Food systems knowledge | N/A | N/A | Observing their caregivers or food worker, and their background knowledge helped improve food systems knowledge in children. | Individual/Household/Organizational | N/A |
| Beck 2021 [12] | Cooking skills | Survey developed by the research team and adapted from existing validated measures | No additional validation for the combined tool | Cooking with grandparents improved children's cooking skills. | Individual/Household/Community | None |
| Beckman 2008 [13] | Nutrition knowledge | Survey developed by the research team | Expert review reported and internal consistency reported; no additional validation conducted | Exposure to nutrition and gardening education with a focus on food system and cooking improved nutrition knowledge more in boys than in girls. | Individual/Community | Age, gender, ethnicity, and program location |
| Bell 2018 [14] | Self-efficacy to eat, cook, and garden fruit and vegetables | Validated existing measures | Validated | After the 3-week intervention, Virtual Sprouts participants significantly improved their self-efficacy to eat, cook, and garden fruit and vegetables. | Individual/Organizational | Age, sex, ethnicity, school, and free school lunch, and total energy intake |
| Binder 2020 [15] | Nutrition knowledge | Existing measure - nutrition knowledge task | Validated | More time spent on media was associated with lower nutrition knowledge in children. Children's age is a positive predictor of children's nutrition knowledge. | Individual/Household | Children's age, sex, and nutrition knowledge at baseline |
| Bisset 2008 [16] | Nutrition knowledge | Survey developed by the research team | Pilot tested; internal consistency reported; no additional validation conducted | Exposure to "Petits cuisots- parents en reseaux" intervention increased children's nutrition knowledge. | Individual/Household/Organizational | Sex, newness to the school, presence of sibling participating in program, and family and/or parental participation in school |
| Blanchet 2020 [17] | Food preparation skills | Self-report questions on frequency on food preparation by trained research assistants | Not validated | Children living in food-insecure households had similar or better food skills than children from food-secure households. Girls living in food-insecure households were less involved in food choices than their food-secure counterparts, whereas boys living in food-insecure households were more involved in family food preparation than boys living in food-secure households. | Individual/Household | Clustering of students in schools, gender, region of residence, number of household residents and parental education attainment |
| Block 2012 [18] | Food preparation skills, and gardening skills | N/A | N/A | Exposure to a program with a focus on learning how to grow, harvest, prepare, share foods significantly increased food preparation skills and gardening skills. | Individual/Organizational | Baseline outcome values, school clustering, and child grade |
| Brennan 2021 [19] | Agri-food knowledge and food label literacy | Agri-food knowledge questionnaire adapted from existing items that were not validated | Not validated | Those in Nourish arm had an increase in agri-food knowledge and understanding of food labels. | Individual/Organizational | Clustering at school level |
| Brown 2020 [20] | Nutrition knowledge | Survey developed by the research team | Not validated | Exposure to nutrition education improved nutrition knowledge in children | Individual/Organizational | None |
| Burrows 2015 [21] | Nutrition knowledge, self-efficacy to cook/consume fruit and vegetables | Survey adapted from previous measure | Internal consistency reported; no additional validation conducted | Cooking sessions together with parental involvement significantly increase children’s nutrition knowledge and self-efficacy to cook/consume fruit and vegetables. | Individual/Household/Organizational | None |
| Caraher 2013 [22] | Cooking confidence | Survey adapted from existing measure | Pilot tested; internal consistency reported; no additional validation conducted | Exposure to a teaching program on nutrition and cooking significantly increased cooking confidence. | Individual/Organizational | None |
| Cason 2001 [23] | Nutrition knowledge | Pictorial knowledge and attitude tool developed by research team | Validated | Exposure to a preschool nutrition education program significantly increased children's nutrition knowledge. | Individual/Organizational | None |
| Castagnoli 2023[24] | Nutrition knowledge | Survey adapted from existing measures (one validated and two others not validated) | No additional validation conducted | Exposure to the intervention improved nutrition knowledge. | Individual/Organizational | None |
| Chen 2014 [25] | Involvement in food preparation | Survey adapted from existing measures | Validated | Children exposed to cooking and tasting activities with parental support showed increased involvement in food preparation at home with their family members. | Individual/Household/Organizational | Ethnicity and grade |
| Chu 2013 [26] | Self-efficacy to select healthy foods | Survey adapted from existing measures | Validated | Involvement in helping with home meal preparation increased children's self-efficacy in selecting healthy foods. | Individual/Household | Gender, household income, parent, education attainment and urban, city or rural residency |
| Colby 2019 [27] | Cooking skills | Survey developed by the research team | Validated | Exposure to cooking with parents or grandparents or another adult children look up to improved children's cooking skills. | Individual/Household/Community | None |
| Colley 2022 [28] | Food and nutrition knowledge | Survey developed by the research team | Not validated | Child sex, race/ethnicity, geographical locations, SES, and parental education influenced food literacy in children. Females had higher food and nutrition scores than males; Caucasian children had higher scores than ethnic minorities; those living in urban areas had lower scores than those living in rural areas. There was no association between age and nutrition knowledge. | Individual/Household/Community | Sex, ethnicity, geographic setting, family income, parent's education |
| Condrasky 2010 [29] | Nutrition knowledge, and cooking skills and confidence | Survey adapted from existing measures | No additional validation for the combined tool conducted | Culinary session on healthy food preparation significantly increased children's nutrition knowledge on food groups, the Food Guide Pyramid; improved cooking skills and increased the confidence in cooking. | Individual/Community | None |
| Cunningham-Sabo 2014 [30] | Cooking self-efficacy | Existing validated measure | Validated | Exposure to nutrition education and cooking activities showed an increase in self-efficacy for food preparation, especially in those who did not cook pre-test. | Individual/Organizational | Gender, prior cooking experience, treatment group |
| Dai 2022 [31] | Food and nutrition knowledge; food preparation skills | Survey developed by the research team | Not validated | Exposure to nutrition education improved food and nutrition knowledge; parent involvement in meal preparation increased home meal preparation in children. | Individual/Household/Organizational | None |
| Dallant 2024 [32] | Food and nutrition knowledge; food literacy | Survey developed by the research team | Validated | Children in fifth grade had a higher level of nutrition knowledge compared to those in fourth grade. Girl had higher food literacy score than boys. | Individual | None |
| Davis 2016 [33] | Nutrition and gardening knowledge, self-efficacy to eat fruit and vegetables, to garden and cook. | Survey developed by the research team | Internal consistency; intra-rater reliability reported; no additional validation conducted | Exposure to the LA Sprouts RCT intervention resulted in increased identification of vegetables and improved nutrition and gardening knowledge but did not result in significant improvements in self-efficacy to eat fruit and vegetables, to garden or cook. | Individual/Organizational | Age, sex, ethnicity, season (Fall, Winter/Spring), schools level, attendance at the intervention classes, English spoken at home, and baseline value for the measure of interest |
| Dawson-McClure 2014 [34] | Nutrition knowledge | Survey developed by the research team - Children's Nutrition and Activity Questionnaire (CNAQ) | Internal consistency; no additional validation conducted | Involving parents in intervention to prevent obesity in children significantly improved child nutrition knowledge. | Individual/Household/Organizational | None |
| Depboylu 2023 [35] | Nutrition literacy | Adolescent Nutrition Literacy Scale (ANLS) | Validated | Increase in physical activity, reduction in screen time, preference for milk/yogurt, no consumption of fast food, drinking water were associated with higher nutrition literacy. No difference between girls' and boys' nutrition literacy were found. | Individual/Household | Gender, age and income level and parent’s education |
| Dixon 2014 [36] | Confidence in meal planning | Survey developed by the research team | Pilot tested; no additional validation conducted | Exposure to meal preparation and cooking techniques with nutrition lessons and menu planning significantly improved confidence in planning a meal. | Individual/Community | None |
| Doustmohammadian 2019 [37] | Food and nutrition literacy | Food and Nutrition literacy (FNLIT) survey developed by the research team | Validated | Child sex, parental education, SES, parental age, and children's birth order influence food and nutrition literacy in children. Girls had higher food choice literacy than boys; boys had a higher level of critical food and nutrition literacy than girls. Higher food and nutrition literacy in children whose mothers' education were higher. Gender, birth order and father's age were important predictors of critical food and nutrition literacy. | Individual/Household | Sex, school status (governmental and nongovernmental), grade, birth rank, family size, ethnicity, parents education, father job position and mother employment, Other income source of family members, house ownership status and financial support source |
| Doustmohammadian 2022 [38] | Food and nutrition literacy | Food and Nutrition literacy (FNLIT) survey developed by the research team | Validated | Small family size, private schooling, mothers’ education, occupation, age and ethnicity, were the most important predictors of food and nutrition literacy. Children with adequate levels of food and nutrition literacy have mothers with high-education levels, employed mothers, small family sizes, and children with private schooling children. | Individual/Household | Age, birth order, family size, ethnicity, parental age, parental education, father’s job position, mother’s employment status, other income source(s) of family members, house ownership status, and receiving financial support |
| Elsborg 2022 [39] | Food and nutrition knowledge; food label literacy; overall food literacy | Food Literacy Questionnaire for children (FLQ-sc) | Validated | Exposure to an intervention on food preparation skills such as cooking, sustainability, and to engage in with multiple dimensions of food had a positive impact on nutrition knowledge and food label literacy. | Individual/Organizational | Age, sex, socio-economic status |
| Ensaff 2017 [40] | Cooking skills | Survey adapted from existing measures | Pilot tested; internal consistency reported; test-retest reliability reported; no additional validation conducted | Exposure to sessions on preparation skills improved cooking skills. | Individual/Organizational | School, intervention, pupil’s age and baseline response |
| Espinosa-Curiel 2020 [41] | Food knowledge | Survey developed by the research team | Not validated | Exposure to the game with nutritional information and behaviour change techniques significantly improved their food knowledge. | Individual/Organizational | None |
| Evans 2012 [42] | Nutrition knowledge; self-efficacy to eat fruit and vegetables | Survey developed by the research team | Not validated | Exposure to intervention classes on local food systems, gardening and food production improved nutrition knowledge and self-efficacy to eat fruit and vegetables. | Individual/Organizational | Gender, ethnicity, and socio-economic status |
| Eves 2010 [43] | Food hygiene and storage knowledge | Activity-tools developed by research team | Not validated | Exposure to activities on food hygiene/storage, improved children's knowledge on food hygiene and storage. | Individual/Organizational | None |
| Frerichs 2016 [44] | Categorization of healthy versus unhealthy foods (Nutrition knowledge) | N/A | N/A | Children's age influenced food literacy - older children were more able to categorize foods into healthy versus healthy (nutrition knowledge). | Individual/Organizational | N/A |
| Friel 1999 [45] | Nutrition knowledge | Validated pictorial survey from Minnesota Heart Health program | Validated | Exposure to the education program showed no improvement in nutrition knowledge. | Individual/Organizational | None |
| Froome 2020 [46] | Nutrition knowledge | Nutrition Attitudes and Knowledge (NAK) Questionnaire | Validated | Exposure to Foodbot Factory significantly improved overall nutrition knowledge. | Individual/Community | None |
| Fulkerson 2010 [47] | Food preparation skills | Survey developed by the research team | Internal consistency reported; no additional validation conducted | Exposure to family meal preparation and interactive nutrition education activities, in both parents and children in the intervention program led to greater food preparation skills in children. | Individual/Household/Community | Baseline level of outcome |
| Gan 2019 [48] | Nutrition knowledge | Survey developed by the research team - Nutrition Knowledge Questionnaire | Validated | Exposure to Healthy Foodie game significantly improved food group (nutrition) knowledge post intervention. | Individual/Organizational | Pretest Food Group Knowledge score and Food Group Knowledge score |
| Gao 2022 [49] | Nutrition knowledge | Existing measure - Nutrition Improvement Program for the Rural Compulsory Education Students was used | Validated | Peer influence, gender, parental dietary and nutrition knowledge, and father's education have a direct impact on students' dietary and nutrition knowledge. Boys had lower nutrition knowledge than girls; parental dietary and nutrition knowledge had a significant impact on children's nutrition knowledge. Fathers’ education had a higher impact on children's nutrition knowledge. | Individual/Household/Interpersonal | Age, gender, parents' age and education, household assets, school effects |
| Gatto 2012 [50] | Self-efficacy to eat and cook fruit and vegetables | Survey adapted from existing validated measures | No additional validation for the combined tool | Educating children on cooking and gardening increased self-efficacy to eat and cook fruit and vegetables. | Individual/Community | Age, sex, and baseline value of the outcome |
| Gunther 2019 [51] | Food preparation skills | Survey adapted from existing measure with reported internal consistency reported | No additional validation conducted | Exposure to planning, cooking and eating healthy meals and snacks significantly improved food preparation skills. Older children (aged 9-10 years old) demonstrated an increase in food prep skills compared to the younger children (4-8 years old). | Individual/Community | Race, household income, cohort, baseline outcome values, |
| Ha 2020 [52] | Food advertising literacy | Survey adapted from existing measure | Not validated | Exposure to food advertising training with parental support improved cognitive and critical thinking towards food advertising. | Individual/Household | None |
| Harley 2018 [53] | Nutrition knowledge and self-efficacy to cook. | Survey adapted from existing measures (not all were validated) | No additional validation for the combined tool | Exposure to culinary training promoting intake of wholefood and plant-based diet did not improve self-efficacy for cooking, but significantly improved nutrition knowledge. | Individual/Organizational | Student ethnicity, grade level, mobility, and free/reduced-price lunch (at school level); attendance rate, sex, and baseline values of all primary and secondary outcomes (at individual level) |
| Heerman 2021 [54] | Cooking self-efficacy | Survey adapted from existing validated measures | No additional validation for the combined tool | Exposure to nutrition education, meal prep and cooking skills improved cooking self-efficacy; girls had higher cooking self-efficacy than boys. | Individual/Community | Baseline outcome score, child age and gender |
| Hermans 2018 [55] | Nutrition knowledge | Survey developed by the research team | Not validated | Exposure to Alien Health improved children's nutrition knowledge. | Individual/Organizational | None |
| Hojer 2021 [56] | Nutrition knowledge; kitchen hygiene knowledge; cooking skills; overall food literacy | Survey developed by the research team | Internal consistency reported; no additional validation conducted | Exposure to the sensory-based course with fish improved knowledge on fish and skills related to fish cooking, improved kitchen hygiene, nutrition knowledge and overall food literacy, especially in girls. | Individual/Organizational | None |
| Hollywood 2022 [57] | Perceived cooking competence | Validated survey - Cooking Competence 11-item scale (CooC11) | Validated | Exposure to cooking intervention through online videos in a home setting with parents involved improved perceived cooking competence in children. | Individual/Household | Baseline perceived cooking competence, age, prior cooking experience, perceived movement competence, post wellbeing, |
| Hyland 2006 [58] | Food preparation skills | N/A | N/A | Food Club is an appropriate and feasible approach to developing food preparation skills in children. | Individual/Organizational | N/A |
| Jacob 2019 [59] | Nutrition knowledge and cooking skills | Survey developed by the research team | Not validated | Exposure to cooking workshops showed no improvement in cooking skills, but in nutrition knowledge. | Individual/Community | Age and gender |
| Jarpe-Ratner 2016 [60] | Nutrition knowledge and cooking self-efficacy | Survey adapted from existing validated measures | No additional validation for the combined tool conducted | Exposure to an after-school program on cooking and nutrition education increased nutrition knowledge and cooking self-efficacy. | Individual/Organizational | None |
| Johnson-Jennings 2020 [61] | Ancestral food knowledge and skills | Survey adapted from existing measure | Not validated | Exposure to the program with parents' involvement in meal prep and a focus on food systems, nutrition education, gardening and increasing food access increased traditional food knowledge and Indigenous relationships to foods. | Individual/Household/Community | None |
| Johnston 2018 [62] | Nutrition knowledge | Survey developed by the research team | Not validated | Exposure to an interactive in-class nutrition education program improved nutrition knowledge. | Individual/Organizational | None |
| Jung 2019 [63] | Food literacy and nutrition knowledge | Survey developed by the research team | Not validated | Exposure to an intervention on food literacy education, cafeteria activities on healthy foods and basic nutrition improved food literacy and nutrition knowledge. | Individual/Organizational | None |
| Kanellopoulou 2021 [64] | Nutrition literacy | Nutrition Literacy scale developed by research team | Validated | Paternal hypertension, parental health status, children's birth order influenced nutrition literacy in children. Paternal hypertension was inversely associated with the level of nutrition literacy of their children. Parental health status, specifically, paternal diabetes and maternal dyslipidemia were associated with children’s higher level of nutrition literacy. There was a negative association between children's nutrition literacy and birth order. First born children had higher nutrition literacy than their siblings. | Individual/Household | Age and gender |
| Karpouzis 2024 [65] | Food and nutrition knowledge; food preparation and cooking skills; food waste behaviours | Survey adapted from existing measure | No additional validation conducted | There was no significant improvement in food and nutrition knowledge, food preparation and cooking skills, and food waste behaviours. | Individual/Organizational | Age, sex, students speaking another, language at home, and school’s Index of Community Socio-Educational Advantage (ICSEA) |
| Katz 2011 [66] | Nutrition knowledge and food label literacy | Survey developed by the research team | Not validated | Exposure to nutrition education with parents' involvement showed a significant improvement in food label literacy and nutrition knowledge. | Individual/Household/Organizational | None |
| Katz 2014 [67] | Food label literacy and nutrition knowledge | Survey developed by the research team | Validated | Exposure to nutrition education and nutrition labelling activities improved food label literacy and nutrition knowledge. | Individual/Organizational | None |
| Kelly 2022 [68] | Nutrition knowledge and skills; food systems knowledge | N/A | N/A | Exposure to food and nutrition education, tasting new foods, and gardening improved nutrition knowledge and skills, and food systems knowledge. | Individual/Organizational | None |
| Khorramrouz 2020 [69] | Food and nutrition literacy | Food and Nutrition literacy (FNLIT) survey developed by the research team | Validated | Food-secure children had higher food and nutrition literacy scores than food-insecure ones. Food insecure children had significantly lower levels of food and nutrition literacy and subscales such as nutrition knowledge, food choice literacy, and food label literacy. | Individual/Household | Sex, grade, BMI, birth order, parents' age and education |
| Knapp 2019 [70] | Nutrition and food-related knowledge and skills. | N/A | N/A | Exposure to a previous interactive kitchen garden program improved nutrition and food-related knowledge and skills | Individual/Organizational | N/A |
| Koch 2006 [71] | Nutrition Knowledge | Survey developed by the research team | Internal consistency reported; no additional validation reported | Exposure to the garden program and healthy eating intervention significantly improved nutrition knowledge. | Individual/Community | None |
| Kocyigit 2025 [72] | Nutrition knowledge | N/A | N/A | Exposure to the intervention improved children's knowledge of MyPlate and they were better at identifying fruits, grains, and protein, as well as healthy vs. unhealthy foods | Individual/Organizational | None |
| Labbé 2023 [73] | Cooking skills and nutrition knowledge | Survey adapted from existing validated measures | Pilot tested; no additional validation reported for the combined tool | Exposure to an intervention focusing on culinary skills, and food and nutrition education improved cooking skills and nutrition knowledge. Boys improved their cooking skills and food knowledge compared to girls. | Individual/Organizational | Age, gender, ethnicity, school, and family socioeconomic status |
| Lakshman 2010 [74] | Nutrition knowledge | Survey developed by the research team | Pilot tested; no additional validation reported | Exposure to program on nutrition education and healthy eating significantly increased nutrition knowledge. | Individual/Organizational | Baseline nutrition knowledge score, school size, and deprivation |
| Liao 2016 [75] | Food advertising literacy and nutrition knowledge | Survey developed by the research team | Validated | Exposure to food advertising literacy and nutrition education showed significant improvement in nutrition knowledge and food advertising literacy. | Individual/Organizational | Pretest scores |
| Liquori 1998 [76] | Nutrition knowledge and self-efficacy to cook | Survey developed by the research team | Pilot tested; internal consistency reported; no additional validation reported | Exposure to school lunch component, cooking workshops with parental involvement and food and environment lessons had a positive effect on nutrition knowledge and cooking self-efficacy. | Individual/Household/Organizational | Pretest scores |
| Liu 2021 [77] | Food and nutrition literacy | Validated Food and Nutrition Literacy Survey for Chinese school-age children (FNLQ-SC) | Validated | Being a girl and an only child, living someplace other than at school, living in urban areas, permanent residence status, being from an affluent family, and being cared for by parents/grandparents with a higher education level, had nutrition education experience in schools influenced food literacy in children. Home food environment such as accessibility of fruits at home were predictors of food and nutrition literacy in school-age children. | Individual/Household/Community | Age, sex, only-child status, registered residence (urban/rural), family affluence status, caregiver education level, home food environment variables (fruit accessibility, watching videos while eating, family nutrition discussions, eating out), and school nutrition education. |
| Mack 2020 [78] | Nutrition knowledge | Survey developed by the research team | Pilot tested; no additional validation conducted | Exposure to the game designed for obesity prevention significantly improved nutrition knowledge. | Individual/Organizational | Cluster randomization |
| Maiz 2021 [79] | Cooking self-efficacy | Previously validated survey | Validated | Children exposed to the intervention HO and NE significantly improved their cooking self-efficacy. | Individual/Organizational | Gender, food neophobia, baseline vegetable liking, and frequency of eating at the school canteen |
| Morgan 2010 [80] | Nutrition knowledge | Survey adapted from existing validated measure | No additional validation conducted | Exposure to nutrition education and gardening with parental support significantly increased fruit and vegetable knowledge. | Individual/Household/Organizational | Pre test scores |
| Morgan 2014 [81] | Nutrition knowledge | Survey adapted from existing validated measure - Knowledge, Attitudes, and Behaviors (KAB) | No additional validation conducted | Exposure to nutrition education with physical activity significantly increased nutrition knowledge. | Individual/Organizational | None |
| Morris 2001 [82] | Nutrition knowledge | Survey developed by the research team | Not validated | Exposure to nutrition education and gardening improved children's nutrition knowledge. | Individual/Organizational | Pre test scores |
| Morris 2002 [83] | Nutrition knowledge | Survey developed by the research team | Reliability reported; No additional validation conducted | Nutrition and gardening lessons with parental involvement significantly increased nutrition knowledge. | Individual/Household/Organizational | Pre test scores |
| Moss 2013 [84] | Nutrition knowledge | Survey adapted from existing measures | No additional validation conducted for combined tool | Exposure to nutrition education classes with a farm tour significantly increased nutrition knowledge in children. | Individual/Organizational | None |
| Murad 2021 [85] | Cooking skills and confidence in making sustainable food choices | Validated Tool for Food Literacy Assessment in Children (TFLAC) | Validated | Exposure to cooking lessons or nutrition education increased confidence in cooking, making sustainable food choices. | Individual/Household | None |
| Muzaffar 2014 [86] | Meal planning skills | Previously validated survey | Validated | After exposure to online educational intervention on diabetes/obesity prevention, children significantly improved their meal planning skills. | Individual/Organizational | None |
| Nelson 2016 [87] | Nutrition knowledge | Survey developed by the research team (Not validated) | Not validated | Exposure to a short-term food-focused advertising literacy school curriculum improved children's nutrition knowledge. | Individual/Organizational | None |
| Ng 2022 [88] | Knowledge and self-efficacy to prepare healthy meals. | Survey developed by the research team | Validated | Exposure to the intervention improved knowledge and self-efficacy of healthy meal preparation. | Individual/Organizational | Baseline scores |
| Nogueira 2022 [89] | Food and nutrition knowledge | Survey developed by the research team | Not validated | Exposure to food literacy and nutrition education program increased children's food and nutrition knowledge. | Individual/Organizational | N/A |
| Nozue 2016 [90] | Cooking skills | Survey adapted from a national survey | Not validated | Children involved in food-related activities group had better cooking skills than children not involved in food-related activities group. | Individual/Household | Prefecture, family structure, and number of siblings |
| O'Brien 2006 [91] | Nutrition knowledge | Survey adapted from existing measures | No additional validation conducted for combined tool | Exposure to both nutrition education and gardening lessons had no effect on nutrition knowledge. | Individual/Organizational | None |
| Olan 2019 [92] | Nutrition Knowledge | Food Fury Quiz (imade-drawing task) | Not validated | Exposure to nutrition education with farm tours increased children’s nutrition knowledge. | Individual/Organizational | None |
| Overcash 2018 [93] | Self-efficacy to cook | Previously validated survey | Validated | Exposure to food preparation with parents and nutrition education significantly improved child self-efficacy toward cooking. | Individual/Household/Community | None |
| Parmer 2009 [94] | Nutrition knowledge | Survey adapted from existing measure | Internal consistency reported; no additional validation reported | Exposure to nutrition education and/or gardening component significantly increased fruit and vegetable knowledge. | Individual/Organizational | None |
| Perez-Rodrigo 1997 [95] | Nutrition knowledge; food preparation skills; kitchen safety knowledge; food label literacy | Survey developed by the research team | Not validated | Exposure to nutrition education with parental involvement, changes to school meals to supply 35% of RDA, consideration to cultural and ethnic differences significantly increase children's knowledge nutrition, improved nutrition knowledge, food preparation skills, and kitchen safety knowledge in the kitchen; scored better in reading food labels. | Individual/Household/Organizational | None |
| Pirouznia 2001 [96] | Nutrition knowledge | Existing measure - Comprehensive assessment of nutrition knowledge, attitudes, and practices (CANKAP) - Validated | Validated | Child's sex had an influence on food literacy - Girls had significantly higher mean nutrition knowledge scores than boys in the 7th and 8th grades, but no significant difference in nutrition knowledge were found for boys and girls in 6th grade. | Individual | None |
| Policastro 2023 [97] | Self-efficacy to cook | Adapted from Tool for Food Literacy Assessment in Children (TFLAC) | No additional validation conducted | Exposure to a culinary literacy program for children improved their self-efficacy to cook. | Individual/Organizational | None |
| Poston 2005 [98] | Nutrition knowledge and gardening skills | Survey questions drawn from previously validated surveys | No additional validation for the combined tool | Exposure to nutrition education with a focus on gardening did not improve nutrition knowledge, but increased gardening skills during the summer months. | Individual/Community | None |
| Powell 2018 [99] | Media and food marketing literacy | N/A | N/A | The media literacy intervention raised awareness where both children and parents learned to be more critical of ads. | Individual/Household/Community | None |
| Powers 2005 [100] | Nutrition knowledge | Survey developed by the research team | Pilot tested; content validity reported; readability and reliability reported; no additional validation | Exposure to nutrition education based on the tenets of SCT significantly improved nutrition knowledge in children. | Individual/Organizational | None |
| Puma 2013 [101] | Nutrition knowledge and food label literacy | Survey developed by the research team and adapted from existing measures | Pilot tested; no additional validation reported | Exposure to nutrition education program grounded on theories significantly increased nutrition knowledge about the Food Guide Pyramid and the number of fruit and vegetables to consume/day and knowledge about which foods are healthier and label reading skills. | Individual/Organizational | None |
| Quinn 2003 [102] | Nutrition knowledge | Survey adapted from existing validated measure in adults | Not validated for use in children | A cooking program with parental involvement on the origins of fruit and vegetables significantly increased children's fruit and vegetables' knowledge. | Individual/Household/Organizational | None |
| Ratcliffe 2011 [103] | Nutrition knowledge | Survey developed by the research team | Pilot tested; no additional validation reported | Exposure to garden-based learning improved students' ability to identify vegetables correctly. | Individual/Organizational | None |
| Rodriguez 2015 [104] | Nutrition knowledge and gardening skills | N/A | N/A | A previous exposure to a school extension garden program, improved children's nutrition knowledge and gardening skills. | Individual/Organizational | N/A |
| Rosi 2016a [105] | Nutrition knowledge and food label literacy | Survey developed by the research team | Not validated | Exposure to nutrition education improved food and nutrition knowledge, but there was not much difference in food label literacy. | Individual/Organizational | None |
| Rosi 2016b [106] | Nutrition knowledge | Survey developed by the research team | Not validated | Intervention focused on nutrition education through games, activities, and humanoid robot that has a background in food science significantly increased nutrition knowledge. | Individual/Organizational | None |
| Sahye-Pudaruth 2024 [107] | Cooking skills | Adapted from Tool for Food Literacy Assessment in Children (TFLAC) | Internal consistency reported; no additional validation reported | Early life involvement in food skills was significantly associated with children's cooking skills | Individual/Household | Child age at follow-up, parent age at baseline, child sex, household income, and intervention status |
| Sahye-Pudaruth 2025 [108] | Cooking skills | Adapted from Tool for Food Literacy Assessment in Children (TFLAC) | Internal consistency reported; no additional validation reported | Although parents possessed relatively good food skills, there was no significant association between their food skills and children's cooking skills. | Individual/Household | Child age and sex, parent age, household income, and intervention status |
| Saksvig 2005 [109] | Nutrition knowledge | Survey adapted by existing measures | No additional validation for combined tool | Exposure to an intervention with parental/peer involvement, and school-wide policy as well as school lunch program significantly improved nutrition knowledge. | Individual/Household/Interpersonal/Organizational | Baseline outcome score, sex, grade level, baseline obesity status, ability to speak Oji-Cree, parent/guardian education level, and parental purchase of low-fat, high-fiber foods |
| Scherr 2017 [110] | Nutrition knowledge | Survey developed by the research team | Not validated | Exposure to garden nutrition education, harvesting, growing produce, cooking demonstrations, sharing of those meals with family, educating parents on positive parenting practices through newsletters, health fairs, installation of salad bars to provide fresh seasonal fruits and vegetables in the lunchroom, school site wellness community to improve school community by applying the district school wellness policies and reinforcing SHCP program objectives significantly improved food and nutrition knowledge. | Individual/Household/Organizational | Race/ethnicity, household income, baseline nutrition knowledge score, Baseline science process skills score, moderate/ vigorous physical activity, and reported parent use of pressure to eat |
| Schmidt 2022 [111] | Nutrition knowledge and culinary skills | Survey adapted from existing measures | Pilot tested; no additional validation reported | Exposure to nutrition education and culinary skills, and guided food tasting showed significant improvements in children’s nutrition knowledge and self-reported culinary skills. | Individual/Community | Within subject correlations (pre/post) and the implementation cycle in which children were enrolled. |
| Scott 2022 [112] | Involvement in cooking | N/A | N/A | Parents spending more time with their children planning meals, shopping, and preparing meals led to improved cooking in children. | Individual/Household | N/A |
| Shannon 1988 [113] | Nutrition knowledge | Survey developed by the research team | Validated | Exposure to nutrition education significantly increased nutrition knowledge. | Individual/Organizational | Pretest scores, time, treatment group |
| Sharkey 2023 [114] | Nutrition knowledge and self-efficacy in food preparation and cooking | Survey developed by the research team | Not validated | Exposure to CwS4H significantly improved nutrition knowledge and self-efficacy in food preparation and cooking | Individual/Community | None |
| Smith 2016 [115] | Nutrition knowledge | Survey developed by the research team | Pilot tested; no additional validation reported | Exposure to an intervention on increasing familiarity with pulses through garden activities significantly increased knowledge on pulses. | Individual/Organizational | None |
| Smolak 1998 [116] | Nutrition knowledge | Survey developed by the research team | Not validated | Exposure to a curriculum on healthy eating and exercise with parental involvement did not significantly increase children’s knowledge on the Food Guide Pyramid. | Individual/Household/Organizational | None |
| Somerset 2009 [117] | Nutrition knowledge | Survey developed by the research team | Pilot tested; test-retest reliability reported; no additional validation reported | Exposure to school-based garden activities increased children’s nutrition knowledge. | Individual/Organizational | None |
| Tabacchi 2020 [118] | Nutrition knowledge | Validated pre-school FLAT (Food Literacy Assessment Tool) | Validated | Female gender, older child age, greater height, lower socio-economic status (SES) were independent predictors of higher food literacy. Children in lower SES had higher knowledge and skills related to traditional foods. | Individual/Household | Gender, age, weight, height, BMI, school, socio-economic environment, gross motor and emergent literacy skills |
| Tabacchi 2021 [119] | Food literacy | Validated pre-school FLAT (Food Literacy Assessment Tool) | Validated | Mothers’ higher education and physical activity were associated with an increased food literacy level. | Individual/Household | Mothers’ education level, income, BMI, physical activity and sedentariety, and breastfeeding duration; child age, food habits, BMI, and physical activity |
| Townsend 2006 [120] | Nutrition knowledge; food preparation skills; food safety practices | Kids Kartoon booklet developed by the research team | Validated | When exposed to an intervention with food preparation and tasting, nutrition knowledge and food preparation skills and food safety practices improved significantly. | Individual/Community | Pretest scores, gender, age, and ethnicity, with group nested in condition |
| Treu 2017 [121] | Food label literacy | Validated FLANK (Food Label Literacy for Applied Nutrition Knowledge) survey | Validated | Exposure to both standard and enhanced interventions with reinforcement messages to parents and demonstrations at supermarkets on healthful food choices significantly improved food label literacy in children. | Individual/Household/Organizational | Not reported |
| Truman 2019 [122] | Media and food marketing literacy and food label literacy | Survey developed by the research team | Not validated | Exposure to media food literacy training showed an increase in understanding food marketing appeals and improved food label literacy. | Individual/Organizational | None |
| Tuuri 2009 [123] | Nutrition knowledge | Survey adapted from existing measures | Validated | Exposure to a program on wellness emphasizing the consumption of fruit and vegetables increased students' nutrition knowledge. | Individual/Organizational | Treatment, test, gender, grade, race/ethnicity, school, and students |
| Varì 2022 [124] | Food and nutrition knowledge; meal planning | Survey developed by the research team | Not validated | Exposure to an intervention focusing on handling and processing of food, functions of nutrients, knowledge about plants, variety and seasonality of vegetables, food waste, environmental footprint, sustainable diets, and cooking at home with family improved nutrition knowledge and children's ability to create weekly menu plans as per nutrition guidelines. | Individual/Household/Organizational | Not reported |
| Varì 2023 [125] | Food and nutrition knowledge; meal planning | Survey developed by the research team | Not validated | Exposure to an intervention focusing on handling and processing of food, functions of nutrients, knowledge about plants, variety and seasonality of vegetables, food waste, environmental footprint, sustainable diets, and cooking at home with family improved nutrition knowledge and children's ability to create weekly menu plans as per nutrition guidelines. | Individual/Household/Organizational | Not reported |
| Vaughan 2025 [126] | Cooking skills, cooking knowledge, and nutrition knowledge, food systems knowledge | Tool for Food Literacy Assessment in Children (TFLAC-UK) - Not validated but adapted from TFLAC | Not validated | Exposure to Phunky Foods did not improve foood literacy (cooking skills, cooking knowledge, and nutrition knowledge, food systems knowledge) | Individual/Organizational | Clustering, baseline value of outcome (food literacy, cooking skills, fruit/vegetable intake), sex, % eligibility for free school meals, school engagement score |
| Velardo 2019 [127] | Food-related and nutrition knowledge; cooking skills; self-efficacy to prepare and choose healthy foods | N/A | N/A | Children's own nutrition knowledge, parents involving children in food shopping, food preparation, food availability, lower SES, nutrition education in school settings, and food marketing campaigns influenced food literacy in children. | Individual/Household/Community/Organizational | N/A |
| de Vlieger 2021 [128] | Nutrition knowledge | Used existing measure - Child Nutrition Knowledge Questionnaire - Australian version (CNK-AU) | Validated | Engagement with VitaVillage significantly improved children’s overall nutrition knowledge | Individual/Organizational | None |
| Weber 2018 [129] | Cooking skills, basic knowledge on kitchen hygiene, and nutrition knowledge | Survey developed by the research team | Not validated | Exposure to nutrition education, food preparation, practical handling of foods and kitchen utensils improved cooking skills, basic kitchen hygiene knowledge and nutrition knowledge. | Individual/Organizational | Baseline outcome values, age at baseline, sex, school, and migration background |
| Wen 2025 [130] | Nutriion literacy | Validated nutrition Literacy Questionnaire for Chinese Pre-school Children (NLQ-PSC) | Validated | Girls, older and overweight children, children with parents who had higher education level and SES had higher nutrition literacy. | Individual/Household | Age, sex, residence/region, parental education level, household income, and child weight status |
| Whiteley 2015 [131] | Nutrition knowledge and overall food literacy | Survey developed by the research team | Not validated | Exposure to intervention that consist of nutrition lessons, sensory activities with vegetables and recipes that children could make with their parents at home improved nutrition knowledge and food literacy. | Individual/Household/Organizational | N/A |
| Wolfe 2018 [132] | Nutrition label reading | Validated EFNEP (Expanded Food and Nutrition Education Program) federal survey | Validated | Exposure to a curriculum on obesity and chronic disease reduction with hands-on experiential learning significantly improved nutrition label reading. | Individual/Organizational | None |
| Woodruff 2013 [133] | Food preparation skills | Survey adapted from existing validated measures | Expert review reported; test-retest reliability reported; no additional validation reported | Children's age, sex, and race influenced food literacy in children. Food preparation skills were not different by sex. White children were more involved in food prep than non-whites. Older children were more involved in food prep than younger children. Children cooked more often with family members | Individual/Household | Sex, grade, and ethnicity |
| Woodruff 2020 [134] | Nutrition knowledge | Survey adapted from existing validated measures | No additional validation for the combined tool | Exposure to food literacy lessons with a centrally procured school snack program to increase intake of fruit and vegetables showed no significant change in children's nutrition knowledge. | Individual/Organizational | None |
| Wright 2012 [135] | Nutrition knowledge and self-efficacy to choose healthy food | Existing measure - Child and Adolescent Trial for Cardiovascular Health After-School Student Questionnaire (ASSQ) | Validated | Nutrition education at school combined with parental involvement significantly increased nutrition knowledge and self-efficacy to choose healthy food choices. | Individual/Household/Organizational | Pre test scores, child’s race, child’s gender, and parental marital status |
| Xu 2024 [136] | Food literacy | Survey adapted from existing measures that were not fully validated | Not validated | Parental food education was significantly associated with children's food literacy. | Individual/Household | Not reported |
| Yoshii 2021 [137] | Cooking self-efficacy | Survey developed by the research team | Not validated | Exposure to hands-on cooking programs in schools can effectively improve children’s cooking self-efficacy and participation in home cooking activities. | Individual/Household/Organizational | None |
| Zahr 2017 [138] | Cooking skills | Adapted from previous survey | Not validated | Exposure to hands-on cooking and tasting program significantly increased cooking skills. | Individual/Organizational | None |
| Zarnowiecki 2012 [139] | Nutrition knowledge | Validated Healthy Food Knowledge Activity (HFKA) questionnaire | Validated | Parents’ nutrition knowledge, SES, education level and attitudes to health were associated with children’s nutrition knowledge. | Individual/Household | Socio-economic status, parent education level, parents' attitudes toward food and health, children’s television viewing, takeaway meal consumption and special dietary requirements |

**References**

1. Adedokun OA, Bastin S, Plonski P, Najor J, Cotterill D: **Outcome evaluation of the super star chef summer youth nutrition education program.** *The Journal of Extension* 2020, **58:**13.

2. Ahmadpour M, Omidvar N, Shakibazadeh E, Doustmohammadian A, Rahimiforoushani A: **Development and evaluation of an intervention to improve food and nutrition literacy among Iranian Kurdish primary school children: An application of intervention mapping approach.** *Frontiers in Public Health* 2023, **10:**1059677.

3. Al Ali N, Arriaga A, Rubio M: **The cognitive and behavioral impact of a culinary education program on schoolchildren.** *Nutrition & Food Science* 2021, **51:**10-29.

4. Amin SA, Panzarella C, Lehnerd M, Cash SB, Economos CD, Sacheck JM: **Identifying food literacy educational opportunities for youth.** *Health Education & Behavior* 2018, **45:**918-925.

5. Anderson A, Porteous L, Foster E, Higgins C, Stead M, Hetherington M, Ha M, Adamson A: **The impact of a school-based nutrition education intervention on dietary intake and cognitive and attitudinal variables relating to fruits and vegetables.** *Public health nutrition* 2005, **8:**650-656.

6. Auld GW, Romaniello C, Heimendinger J, Hambidge C, Hambidge M: **Outcomes from a school-based nutrition education program alternating special resource teachers and classroom teachers.** *J Sch Health* 1999, **69:**403-408.

7. Austin EW, Austin BW, French BF, Cohen MA: **The effects of a nutrition media literacy intervention on parents’ and youths’ communication about food.** *Journal of Health Communication* 2018, **23:**190-199.

8. Austin EW, Austin B, Kaiser CK, Edwards Z, Parker L, Power TG: **A media literacy-based nutrition program fosters parent–child food marketing discussions, improves home food environment, and youth consumption of fruits and vegetables.** *Childhood Obesity* 2020, **16:**S-33-S-43.

9. Bai Y, Kim Y-H, Han Y-H, Hyun T: **Impact of a school-based culinary nutrition education program on vegetable consumption behavior, intention, and personal factors among Korean second-graders.** *Nutrition Research and Practice* 2018, **12:**527.

10. Baños R, Cebolla A, Oliver E, Alcañiz M, Botella C: **Efficacy and acceptability of an Internet platform to improve the learning of nutritional knowledge in children: the ETIOBE mates.** *Health education research* 2013, **28:**234-248.

11. Barton A, Koch PD, Contento IR, Hagiwara S: **From global sustainability to inclusive education: Understanding urban children’s ideas about the food system.** *International Journal of Science Education* 2005, **27:**1163-1186.

12. Beck AM, Nielsen SB, Bjørnsbo KS: **Grandchildren’s food workshop: Impact of an intergenerational cooking program on dietary habits, food courage, cooking skills and two-way interaction in Danish children and their grandparents.** *Nutrition and health* 2021, **27:**413-421.

13. Beckman LL, Smith C: **An Evaluation of Inner-City Youth Garden Program Participants' Dietary Behavior and Garden and Nutrition Knowledge.** *Journal of Agricultural Education* 2008, **49:**11-24.

14. Bell BM, Martinez L, Gotsis M, Lane HC, Davis JN, Antunez-Castillo L, Ragusa G, Spruijt-Metz D: **Virtual Sprouts: A Virtual Gardening Pilot Intervention Increases Self-Efficacy to Cook and Eat Fruits and Vegetables in Minority Youth.** *Games Health J* 2018, **7:**127-135.

15. Binder A, Naderer B, Matthes J, Spielvogel I: **Fiction Is Sweet. The impact of media consumption on the development of children’s nutritional knowledge and the moderating role of parental food-related mediation. A longitudinal study.** *Nutrients* 2020, **12:**1478.

16. Bisset SL, Potvin L, Daniel M, Paquette M: **Assessing the impact of the primary school-based nutrition intervention Petits cuistots--parents en réseaux.** *Can J Public Health* 2008, **99:**107-113.

17. Blanchet R, Loewen OK, Godrich SL, Willows N, Veugelers P: **Exploring the association between food insecurity and food skills among school-aged children.** *Public Health Nutrition* 2020, **23:**2000-2005.

18. Block K, Gibbs L, Staiger PK, Gold L, Johnson B, Macfarlane S, Long C, Townsend M: **Growing community: the impact of the Stephanie Alexander Kitchen Garden Program on the social and learning environment in primary schools.** *Health Education & Behavior* 2012, **39:**419-432.

19. Brennan SF, Lavelle F, Moore SE, Dean M, McKinley MC, McCole P, Hunter RF, Dunne L, O’Connell NE, Cardwell CR: **Food environment intervention improves food knowledge, wellbeing and dietary habits in primary school children: Project Daire, a randomised-controlled, factorial design cluster trial.** *International Journal of Behavioral Nutrition and Physical Activity* 2021, **18:**1-18.

20. Brown JM, Savaglio R, Watson G, Kaplansky A, LeSage A, Hughes J, Kapralos B, Arcand J: **Optimizing Child Nutrition Education With the Foodbot Factory Mobile Health App: Formative Evaluation and Analysis.** *JMIR Form Res* 2020, **4:**e15534.

21. Burrows TL, Lucas H, Morgan PJ, Bray J, Collins CE: **Impact evaluation of an after-school cooking skills program in a disadvantaged community: back to basics.** *Canadian Journal of Dietetic Practice and Research* 2015, **76:**126-132.

22. Caraher M, Seeley A, Wu M, Lloyd S: **When chefs adopt a school? An evaluation of a cooking intervention in English primary schools.** *Appetite* 2013, **62:**50-59.

23. Cason KL: **Evaluation of a preschool nutrition education program based on the theory of multiple intelligences.** *Journal of nutrition education* 2001, **33:**161-164.

24. Castagnoli JdL, Santos EFd, Novello D: **How Interdisciplinary Interventions Can Improve the Educational Process of Children Regarding the Nutritional Labeling of Foods.** *Foods* 2023, **12:**4290.

25. Chen Q, Goto K, Wolff C, Bianco-Simeral S, Gruneisen K, Gray K: **Cooking up diversity. Impact of a multicomponent, multicultural, experiential intervention on food and cooking behaviors among elementary-school students from low-income ethnically diverse families.** *Appetite* 2014, **80:**114-122.

26. Chu YL, Farmer A, Fung C, Kuhle S, Storey KE, Veugelers PJ: **Involvement in home meal preparation is associated with food preference and self-efficacy among Canadian children.** *Public Health Nutrition* 2013, **16:**108-112.

27. Colby S, Moret L, Olfert MD, Kattelmann K, Franzen-Castle L, Riggsbee K, Payne M, Ellington A, Springer C, Allison C: **Incorporating technology into the iCook 4-H program, a cooking intervention for adults and children: randomized controlled trial.** *JMIR Pediatrics and Parenting* 2019, **2:**e11235.

28. Colley P, Seabrook JA, Woodruff SJ, Gilliland J: **Examining Elementary School Children’s Knowledge about Food and Nutrition in Southwestern Ontario, Canada.** *Canadian Journal of Dietetic Practice and Research* 2022, **83:**59-67.

29. Condrasky MD, Corr AQ, Sharp J, Hegler M, Warmin A: **Culinary nutrition camp for adolescents assisted by dietetic student counselors.** *Topics in Clinical Nutrition* 2010, **25:**362-370.

30. Cunningham-Sabo L, Lohse B: **Impact of a school-based cooking curriculum for fourth-grade students on attitudes and behaviors is influenced by gender and prior cooking experience.** *Journal of nutrition education and behavior* 2014, **46:**110-120.

31. Dai C-L, Chen C-Y, Wang Z, Chang C-L: **Always Open, Seven-Eleven: Education Targeting Healthier Food Choices in a High Convenience Store Density Area in Taipei.** *International Journal* 2022, **11:**73.

32. Dallant T, Bozonnet A, Delarocque-Astagneau E, Gautier S, Koné A, Grasteau V, Rouquette A, Herr M: **Development and evaluation of a food literacy questionnaire for schoolchildren in France.** *Appetite* 2024, **199:**107420.

33. Davis JN, Martinez LC, Spruijt-Metz D, Gatto NM: **LA Sprouts: A 12-Week Gardening, Nutrition, and Cooking Randomized Control Trial Improves Determinants of Dietary Behaviors.** *J Nutr Educ Behav* 2016, **48:**2-11.e11.

34. Dawson-McClure S, Brotman LM, Theise R, Palamar JJ, Kamboukos D, Barajas RG, Calzada EJ: **Early childhood obesity prevention in low-income, urban communities.** *Journal of prevention & intervention in the community* 2014, **42:**152-166.

35. Depboylu GY, Kaner G, Süer M, Kanyilmaz M, Alpan D: **Nutrition literacy status and its association with adherence to the Mediterranean diet, anthropometric parameters and lifestyle behaviours among early adolescents.** *Public Health Nutrition* 2023, **26:**2108-2117.

36. Dixon E, Condrasky MD, Corr A, Kemper K, Sharp J: **Application of a menu-planning template as a tool for promoting healthy preadolescent diets.** *Topics in Clinical Nutrition* 2014, **29:**47-56.

37. Doustmohammadian A, Mohammadi NK, Omidvar N, Amini M, Abdollahi M, Eini-Zinab H, Amirhamidi Z, Esfandiari S, Nutbeam D: **Food and nutrition literacy (FNLIT) and its predictors in primary schoolchildren in Iran.** *Health Promotion International* 2019, **34:**1002-1013.

38. Doustmohammadian A, Omidvar N, Keshavarz-Mohammadi N, Eini-Zinab H, Amini M, Abdollahi M: **The association and mediation role of Food and Nutrition Literacy (FNLIT) with eating behaviors, academic achievement and overweight in 10-12 years old students: a structural equation modeling.** *Nutrition Journal* 2022, **21**.

39. Elsborg P, Thorsen AV, Ravn-Haren G, Bonde AH, Andersen SG, Vermund MC, Klinker CD, Stjernqvist NW: **Improved food literacy among schoolchildren as an effect of a food camp intervention: Results of a controlled effectiveness trial.** *Appetite* 2022, **169:**105845.

40. Ensaff H, Crawford R, Russell J, Barker M: **Preparing and sharing food: a quantitative analysis of a primary school-based food intervention.** *Journal of Public Health* 2017, **39:**567-573.

41. Espinosa-Curiel IE, Pozas-Bogarin EE, Lozano-Salas JL, Martínez-Miranda J, Delgado-Pérez EE, Estrada-Zamarron LS: **Nutritional Education and Promotion of Healthy Eating Behaviors Among Mexican Children Through Video Games: Design and Pilot Test of FoodRateMaster.** *JMIR Serious Games* 2020, **8:**e16431.

42. Evans A, Ranjit N, Rutledge R, Medina J, Jennings R, Smiley A, Stigler M, Hoelscher D: **Exposure to multiple components of a garden-based intervention for middle school students increases fruit and vegetable consumption.** *Health promotion practice* 2012, **13:**608-616.

43. Eves A, Bielby G, Egan B, Lumbers M, Raats M, Adams M: **Food safety knowledge and behaviours of children (5-7 years).** *Health Education Journal* 2010, **69:**21-30.

44. Frerichs L, Intolubbe-Chmil L, Brittin J, Teitelbaum K, Trowbridge M, Huang TTK: **Children's Discourse of Liked, Healthy, and Unhealthy Foods.** *Journal of the Academy of Nutrition and Dietetics* 2016, **116:**1323-1331.

45. Friel S, Kelleher C, Campbell P, Nolan G: **Evaluation of the nutrition education at primary school (NEAPS) programme.** *Public health nutrition* 1999, **2:**549-555.

46. Froome HM, Townson C, Rhodes S, Franco-Arellano B, LeSage A, Savaglio R, Brown JM, Hughes J, Kapralos B, Arcand J: **The Effectiveness of the Foodbot Factory Mobile Serious Game on Increasing Nutrition Knowledge in Children.** *Nutrients* 2020, **12**.

47. Fulkerson JA, Rydell S, Kubik MY, Lytle L, Boutelle K, Story M, Neumark‐Sztainer D, Dudovitz B, Garwick A: **Healthy Home Offerings via the Mealtime Environment (HOME): feasibility, acceptability, and outcomes of a pilot study.** *Obesity* 2010, **18:**S69-S74.

48. Gan FR, Cunanan E, Castro R: **Effectiveness of Healthy Foodie Nutrition Game Application as Reinforcement Intervention to Previous Standard Nutrition Education of School-Aged Children: A Randomized Controlled Trial.** *J ASEAN Fed Endocr Soc* 2019, **34:**144-152.

49. Gao L, Li RT, Zhao PY, Zhang Y: **The Peer Effect on Dietary and Nutritional Cognition among Primary School Students.** *International Journal of Environmental Research and Public Health* 2022, **19**.

50. Gatto NM, Ventura EE, Cook LT, Gyllenhammer LE, Davis JN: **LA Sprouts: a garden-based nutrition intervention pilot program influences motivation and preferences for fruits and vegetables in Latino youth.** *Journal of the Academy of Nutrition and Dietetics* 2012, **112:**913-920.

51. Gunther C, Rogers C, Holloman C, Hopkins LC, Anderson SE, Miller CK, Copeland KA, Dollahite JS, Pratt KJ, Webster A, et al: **Child diet and health outcomes of the simple suppers program: a 10-week, 2-group quasi-experimental family meals trial.** *Bmc Public Health* 2019, **19**.

52. Ha O-R, Killian HJ, Davis AM, Lim S-L, Bruce JM, Sotos JJ, Nelson SC, Bruce AS: **Promoting Resilience to Food Commercials Decreases Susceptibility to Unhealthy Food Decision-Making.** *Frontiers in psychology* 2020, **11:**599663-599663.

53. Harley A, Lemke M, Brazauskas R, Carnegie NB, Bokowy L, Kingery L: **Youth chef academy: Pilot results from a plant‐based culinary and Nutrition literacy program for sixth and seventh graders.** *Journal of School Health* 2018, **88:**893-902.

54. Heerman WJ, Elsakary Y, Sommer EC, Escarfuller J, Barkin SL: **Assessing the scale and spread of an experiential teaching kitchen in after-school programming among school-age children.** *Public Health Nutrition* 2021, **24:**3937-3944.

55. Hermans RCJ, van den Broek N, Nederkoorn C, Otten R, Ruiter ELM, Johnson-Glenberg MC: **Feed the Alien! The Effects of a Nutrition Instruction Game on Children's Nutritional Knowledge and Food Intake.** *Games Health J* 2018, **7:**164-174.

56. Hojer R, Wistoft K, Frost MB: **Yes I can cook a fish; effects of a five week sensory-based experiential theme course with fish on 11-to 13-year old children's food literacy and fish eating behaviour-A quasi-experimental study.** *Food Quality and Preference* 2021, **92**.

57. Hollywood L, Issartel J, Gaul D, McCloat A, Mooney E, Collins CE, Lavelle F: **Cook like a Boss Online: an adapted intervention during the COVID-19 pandemic that effectively improved children’s perceived cooking competence, movement competence and wellbeing.** *International Journal of Behavioral Nutrition and Physical Activity* 2022, **19:**146.

58. Hyland R, Stacy R, Adamson A, Moynihan P: **Nutrition-related health promotion through an after-school project: the responses of children and their families.** *Social Science & Medicine* 2006, **62:**758-768.

59. Jacob R, Motard-Bélanger A, Provencher V, Fernandez MA, Gayraud H, Drapeau V: **Influence of cooking workshops on cooking skills and knowledge among children attending summer day camps.** *Canadian Journal of Dietetic Practice and Research* 2019, **81:**86-90.

60. Jarpe-Ratner E, Folkens S, Sharma S, Daro D, Edens NK: **An experiential cooking and nutrition education program increases cooking self-efficacy and vegetable consumption in children in grades 3–8.** *Journal of nutrition education and behavior* 2016, **48:**697-705. e691.

61. Johnson-Jennings M, Paul K, Olson D, LaBeau M, Jennings D: **Ode'imin Giizis: proposing and piloting gardening as an indigenous childhood health intervention.** *Journal of health care for the poor and underserved* 2020, **31:**871-888.

62. Johnston B, El-Arabi A, Tuomela K, Nelson D: **The Food Doctors: A pilot study to connect urban children and medical students using nutrition education.** *Health Education Journal* 2018, **78:**441-450.

63. Jung T, Huang J, Eagan L, Oldenburg D: **Influence of school-based nutrition education program on healthy eating literacy and healthy food choice among primary school children.** *International Journal of Health Promotion and Education* 2019, **57:**67-81.

64. Kanellopoulou A, Katelari A, Notara V, Antonogeorgos G, Rojas-Gil AP, Kornilaki EN, Kosti RI, Lagiou A, Panagiotakos DB: **Parental health status in relation to the nutrition literacy level of their children: Results from an epidemiological study in 1728 Greek students.** *Mediterranean Journal of Nutrition and Metabolism* 2021, **14:**57-67.

65. Karpouzis F, Lindberg R, Walsh A, Shah S, Abbott G, Ball K: **Impact and process evaluation of a primary-school Food Education and Sustainability Training (FEAST) program in 10-12-year-old children in Australia: pragmatic cluster non-randomized controlled trial.** *BMC Public Health* 2024, **24:**657.

66. Katz DL, Katz CS, Treu JA, Reynolds J, Njike V, Walker J, Smith E, Michael J: **Teaching healthful food choices to elementary school students and their parents: the Nutrition Detectives™ program.** *J Sch Health* 2011, **81:**21-28.

67. Katz DL, Treu JA, Ayettey RG, Kavak Y, Katz CS, Njike V: **Testing the effectiveness of an abbreviated version of the Nutrition Detectives program.** *Prev Chronic Dis* 2014, **11:**E57.

68. Kelly RK, Peralta L, Nash R: **Promoting food literacy in primary school classrooms through the HealthLit4Kids Program in Australia.** *Health Promotion International* 2022, **37:**daac166.

69. Khorramrouz F, Doustmohammadian A, Eslami O, Khadem-Rezaiyan M, Pourmohammadi P, Amini M, Khosravi M: **Relationship between household food insecurity and food and nutrition literacy among children of 9–12 years of age: a cross-sectional study in a city of Iran.** *BMC Research Notes* 2020, **13**.

70. Knapp MB, Hall MT, Mundorf AR, Partridge KL, Johnson CC: **Perceptions of School-Based Kitchen Garden Programs in Low-Income, African American Communities.** *Health Promot Pract* 2019, **20:**667-674.

71. Koch S, Waliczek TM, Zajicek JM: **The effect of a summer garden program on the nutritional knowledge, attitudes, and behaviors of children.** *HortTechnology* 2006, **16:**620-625.

72. Kocyigit E, Bozkurt O, Kocaadam-Bozkurt B, Us Altay D: **Impact of MyPlate and the healthy highway program-based nutrition education intervention on preschoolers' knowledge and food choice.** *BMC Public Health* 2025, **25:**3929.

73. Labbé C, Chiasson SW, Dupuis JB, Johnson C: **Effectiveness of a School-Based Culinary Programme on 9-and 10-Year-Old Children's Food Literacy and Vegetable, Fruit, and Breakfast Consumption.** *Nutrients* 2023, **15**.

74. Lakshman RR, Sharp SJ, Ong KK, Forouhi NG: **A novel school-based intervention to improve nutrition knowledge in children: cluster randomised controlled trial.** *BMC Public Health* 2010, **10:**1-9.

75. Liao LL, Lai IJ, Chang LC, Lee CK: **Effects of a food advertising literacy intervention on Taiwanese children's food purchasing behaviors.** *Health Educ Res* 2016, **31:**509-520.

76. Liquori T, Koch PD, Contento IR, Castle J: **The cookshop program: outcome evaluation of a nutrition education program linking lunchroom food experiences with classroom cooking experiences.** *Journal of Nutrition Education* 1998, **30:**302-313.

77. Liu T, Su X, Li N, Sun J, Ma G, Zhu W: **Development and validation of a food and nutrition literacy questionnaire for Chinese school-age children.** *PLoS One* 2021, **16:**e0244197.

78. Mack I, Reiband N, Etges C, Eichhorn S, Schaeffeler N, Zurstiege G, Gawrilow C, Weimer K, Peeraully R, Teufel M, et al: **The Kids Obesity Prevention Program: Cluster Randomized Controlled Trial to Evaluate a Serious Game for the Prevention and Treatment of Childhood Obesity.** *J Med Internet Res* 2020, **22:**e15725.

79. Maiz E, Urkia-Susin I, Urdaneta E, Allirot X: **Child Involvement in Choosing a Recipe, Purchasing Ingredients, and Cooking at School Increases Willingness to Try New Foods and Reduces Food Neophobia.** *J Nutr Educ Behav* 2021, **53:**279-289.

80. Morgan PJ, Warren JM, Lubans DR, Saunders KL, Quick GI, Collins CE: **The impact of nutrition education with and without a school garden on knowledge, vegetable intake and preferences and quality of school life among primary-school students.** *Public health nutrition* 2010, **13:**1931-1940.

81. Morgan AZ, Ulrich P, Simmons KP, Gropper SS, Connell LJ, Daniels MK, Latham E, Keiley MK: **Effectiveness of a multi-faceted, school-based health intervention program with 4th graders in Alabama.** *Children and Youth Services Review* 2014, **37:**46-54.

82. Morris JL, Neustadter A, Zidenberg-Cherr S: **First-grade gardeners more likely to taste vegetables.** *California Agriculture* 2001, **55**.

83. Morris JL, Zidenberg-Cherr S: **Garden-enhanced nutrition curriculum improves fourth-grade school children's knowledge of nutrition and preferences for some vegetables.** *American Dietetic Association Journal of the American Dietetic Association* 2002, **102:**91-93.

84. Moss A, Smith S, Null D, Long Roth S, Tragoudas U: **Farm to school and nutrition education: positively affecting elementary school-aged children's nutrition knowledge and consumption behavior.** *Childhood obesity* 2013, **9:**51-56.

85. Murad M, Alford A-M, Davis K: **Farm to Future: A Virtual Summer Nutrition Culinary Camp for Kids.** *Journal of Nutrition Education and Behavior* 2021, **53:**445-448.

86. Muzaffar H, Castelli DM, Scherer J, Chapman-Novakofski K: **The impact of web-based HOT (Healthy Outcomes for Teens) Project on risk for type 2 diabetes: a randomized controlled trial.** *Diabetes Technol Ther* 2014, **16:**846-852.

87. Nelson MR, Kehr DP: **Food-Focused Advertising Literacy Can Increase Nutrition Knowledge in Elementary School Students.** *Journal of Nutrition Education and Behavior* 2016, **48:**749-751.e741.

88. Ng CM, Kaur S, Koo HC, Mukhtar F, Yim HS: **Culinary Nutrition Education Improves Home Food Availability and Psychosocial Factors Related to Healthy Meal Preparation Among Children.** *J Nutr Educ Behav* 2022, **54:**100-108.

89. Nogueira T, Ferreira RJ, Sócrates M, Dias da Silva V, Liñan Pinto M, Borrego R, Sousa J: **Sintra Grows Healthy: development and implementation of a food literacy curriculum for primary schools.** *Public Health Nutrition* 2022, **25:**1176-1182.

90. Nozue M, Ishida H, Hazano S, Nakanishi A, Yamamoto T, Abe A, Nishi N, Yokoyama T, Murayama N: **Associations between Japanese schoolchildren's involvement in at-home meal preparation, their food intakes, and cooking skills.** *nrp* 2016, **10:**359-363.

91. O'Brien SA, Shoemaker CA: **An after-school gardening club to promote fruit and vegetable consumption among fourth grade students: the assessment of social cognitive theory constructs.** *Horttechnology* 2006, **16:**24-29.

92. Olan EL, Campbell LO, Jahani S: **Examining Second Graders' Healthy Food Choices: Through Literacy and Active Learning.** *Nutr Metab Insights* 2019, **12:**1178638819839063.

93. Overcash F, Ritter A, Mann T, Mykerezi E, Redden J, Rendahl A, Vickers Z, Reicks M: **Impacts of a Vegetable Cooking Skills Program Among Low-Income Parents and Children.** *J Nutr Educ Behav* 2018, **50:**795-802.

94. Parmer SM, Salisbury-Glennon J, Shannon D, Struempler B: **School gardens: an experiential learning approach for a nutrition education program to increase fruit and vegetable knowledge, preference, and consumption among second-grade students.** *J Nutr Educ Behav* 2009, **41:**212-217.

95. Perez-Rodrigo C, Aranceta J: **Nutrition Education for Schoolchildren Living in a Low-Income Urban Area in Spain.** *Journal of Nutrition Education* 1997, **29:**267-273.

96. Pirouznia M: **The association between nutrition knowledge and eating behavior in male and female adolescents in the US.** *Int J Food Sci Nutr* 2001, **52:**127-132.

97. Policastro P, Brown AH, Comollo E: **Healthy helpers: using culinary lessons to improve children's culinary literacy and self-efficacy to cook.** *Front Public Health* 2023, **11:**1156716.

98. Poston SA, Shoemaker CA, Dzewaltowski DA: **A Comparison of a Gardening and Nutrition Program with a Standard Nutrition Program in an Out-of-school Setting.** *HortTechnology horttech* 2005, **15:**463-467.

99. Powell RM, Gross T: **Food for thought: A novel media literacy intervention on food advertising targeting young children and their parents.** *Journal of Media Literacy Education* 2018, **10:**80-94.

100. Powers AR, Struempler BJ, Guarino A, Parmer SM: **Effects of a nutrition education program on the dietary behavior and nutrition knowledge of second-grade and third-grade students.** *J Sch Health* 2005, **75:**129-133.

101. Puma J, Romaniello C, Crane L, Scarbro S, Belansky E, Marshall JA: **Long-term student outcomes of the Integrated Nutrition and Physical Activity Program.** *J Nutr Educ Behav* 2013, **45:**635-642.

102. Quinn LJ, Horacek TM, Castle J: **The impact of CookshopTM on the dietary habits and attitudes of fifth graders.** *Topics in Clinical Nutrition* 2003, **18:**42-48.

103. Ratcliffe MM, Merrigan KA, Rogers BL, Goldberg JP: **The effects of school garden experiences on middle school-aged students' knowledge, attitudes, and behaviors associated with vegetable consumption.** *Health Promot Pract* 2011, **12:**36-43.

104. Rodriguez MT, Lamm AJ, Odera E, Owens C: **Evaluating impacts of school-based extension garden programs from a child's perspective.** *The Journal of Extension* 2015, **53:**21.

105. Rosi A, Brighenti F, Finistrella V, Ingrosso L, Monti G, Vanelli M, Vitale M, Volta E, Scazzina F: **Giocampus school: a "learning through playing" approach to deliver nutritional education to children.** *Int J Food Sci Nutr* 2016, **67:**207-215.

106. Rosi A, Dall'Asta M, Brighenti F, Del Rio D, Volta E, Baroni I, Nalin M, Coti Zelati M, Sanna A, Scazzina F: **The use of new technologies for nutritional education in primary schools: a pilot study.** *Public Health* 2016, **140:**50-55.

107. Sahye-Pudaruth S, Ma DWL, Prashad M, Haines J: **Early life involvement in food skills is associated with children's cooking skills: a longitudinal analysis.** *Appl Physiol Nutr Metab* 2024.

108. Sahye-Pudaruth S, Ma DWL, Duncan AM, Prashad M, Laila A, Haines J: **Longitudinal associations between mothers’ and fathers’ food skills and their children's cooking skills.** *Applied Physiology, Nutrition, and Metabolism* 2025, **50:**1-7.

109. Saksvig BI, Gittelsohn J, Harris SB, Hanley AJ, Valente TW, Zinman B: **A pilot school-based healthy eating and physical activity intervention improves diet, food knowledge, and self-efficacy for native Canadian children.** *J Nutr* 2005, **135:**2392-2398.

110. Scherr RE, Linnell JD, Dharmar M, Beccarelli LM, Bergman JJ, Briggs M, Brian KM, Feenstra G, Hillhouse JC, Keen CL, et al: **A Multicomponent, School-Based Intervention, the Shaping Healthy Choices Program, Improves Nutrition-Related Outcomes.** *J Nutr Educ Behav* 2017, **49:**368-379.e361.

111. Schmidt S, Goros MW, Gelfond JAL, Bowen K, Guttersen C, Messbarger-Eguia A, Feldmann SM, Ramirez AG: **Children's Afterschool Culinary Education Improves Eating Behaviors.** *Front Public Health* 2022, **10:**719015.

112. Scott L, Ensaff H: **COVID-19 and the National Lockdown: How Food Choice and Dietary Habits Changed for Families in the United Kingdom.** *Front Nutr* 2022, **9:**847547.

113. Shannon B, Chen AN: **A three-year school-based nutrition education study.** *Journal of Nutrition Education* 1988, **20:**114-124.

114. Sharkey JR, Smith A: **Cooking with the Seasons for Health (CwS4H): An Innovative Intervention That Links Nutrition Education, Cooking Skills, and Locally Grown Produce to Increase Vegetable Intake among Limited-Resource Parent-Child Dyads in Rural Washington.** *Nutrients* 2023, **15**.

115. Smith DK, Mandal B, Wallace ML, Riddle LA, Kerr S, Atterberry KA, Miles CA: **Exploring Pulses through Math, Science, and Nutrition Activities.** *The Journal of Child Nutrition & Management* 2016, **40**.

116. Smolak L, Levine MP, Schermer F: **A controlled evaluation of an elementary school primary prevention program for eating problems.** *J Psychosom Res* 1998, **44:**339-353.

117. Somerset S, Markwell K: **Impact of a school-based food garden on attitudes and identification skills regarding vegetables and fruit: a 12-month intervention trial.** *Public Health Nutr* 2009, **12:**214-221.

118. Tabacchi G, Battaglia G, Alesi M, Paoli A, Palma A, Bellafiore M: **Food literacy predictors and associations with physical and emergent literacy in pre-schoolers: Results from the Training-to-Health Project.** *Public health nutrition* 2020, **23:**356-365.

119. Tabacchi G, Petrigna L, Battaglia G, Navarra G, Palma A, Bellafiore M: **An Interaction Path of Mothers' and Preschoolers' Food- and Physical Activity-Related Aspects in Disadvantaged Sicilian Urban Areas.** *Int J Environ Res Public Health* 2021, **18**.

120. Townsend MS, Johns M, Shilts MK, Farfan-Ramirez L: **Evaluation of a USDA nutrition education program for low-income youth.** *J Nutr Educ Behav* 2006, **38:**30-41.

121. Treu JA, Doughty K, Reynolds JS, Njike VY, Katz DL: **Advancing School and Community Engagement Now for Disease Prevention (ASCEND).** *Am J Health Promot* 2017, **31:**143-152.

122. Truman E, Elliott C: **Health-promoting skills for children: Evaluating the influence of a media literacy and food marketing intervention.** *Health Education Journal* 2019, **79:**431-445.

123. Tuuri G, Zanovec M, Silverman L, Geaghan J, Solmon M, Holston D, Guarino A, Roy H, Murphy E: **"Smart Bodies" school wellness program increased children's knowledge of healthy nutrition practices and self-efficacy to consume fruit and vegetables.** *Appetite* 2009, **52:**445-451.

124. Varì R, d'Amore A, Silenzi A, Chiarotti F, Del Papa S, Giovannini C, Scazzocchio B, Masella R: **Improving Nutrition Knowledge and Skills by the Innovative Education Program MaestraNatura in Middle School Students of Italy.** *Nutrients* 2022, **14**.

125. Varì R, Silenzi A, d'Amore A, Catena A, Masella R, Scazzocchio B: **MaestraNatura Reveals Its Effectiveness in Acquiring Nutritional Knowledge and Skills: Bridging the Gap between Girls and Boys from Primary School.** *Nutrients* 2023, **15**.

126. Vaughan KL, Vidal M, Cade JE, Hetherington MM, Evans CEL: **Evaluation of the school-based 'PhunkyFoods' intervention: a cluster randomised controlled trial in the UK.** *Public Health Nutr* 2025, **28:**e86.

127. Velardo S, Drummond M: **Qualitative insight into primary school children’s nutrition literacy.** *Health Education* 2019, **119:**98-114.

128. de Vlieger NM, Sainsbury L, Smith SP, Riley N, Miller A, Collins CE, Bucher T: **Feasibility and Acceptability of 'VitaVillage': A Serious Game for Nutrition Education.** *Nutrients* 2021, **14**.

129. Weber KS, Eitner J, Dauben L, Spörkel O, Strassburger K, Sommer J, Kaiser B, Buyken AE, Kronsbein P, Müssig K: **Positive Effects of Practical Nutrition Lessons in a Primary School Setting with a High Proportion of Migrant School Children.** *Exp Clin Endocrinol Diabetes* 2020, **128:**111-118.

130. Wen J, Zhang X, Yin X, Ma G, Wang J: **Development and Validation of Nutrition Literacy Questionnaire for Chinese Pre-School Children.** *Nutrients* 2025, **17:**1704.

131. Whiteley C, Matwiejczyk L: **Preschool Program Improves Young Children’s Food Literacy and Attitudes to Vegetables.** *Journal of Nutrition Education and Behavior* 2015, **47:**397-398.e391.

132. Wolfe WS, Scott-Pierce M, Dollahite J: **Choose Health: Food, Fun, and Fitness Youth Curriculum Promotes Positive Behaviors.** *J Nutr Educ Behav* 2018, **50:**924-930.

133. Woodruff SJ, Kirby AR: **The Associations Among Family Meal Frequency, Food Preparation Frequency, Self-efficacy for Cooking, and Food Preparation Techniques in Children and Adolescents.** *Journal of Nutrition Education and Behavior* 2013, **45:**296-303.

134. Woodruff SJ, Beckford C, Segave S: **Fruit and Vegetable Lesson Plan Pilot Intervention for Grade 5 Students from Southwestern Ontario.** *Int J Environ Res Public Health* 2020, **17**.

135. Wright K, Norris K, Newman Giger J, Suro Z: **Improving healthy dietary behaviors, nutrition knowledge, and self-efficacy among underserved school children with parent and community involvement.** *Child Obes* 2012, **8:**347-356.

136. Xu X, Cai H, Zhang J, Xia T: **The Effects of Parental Food Education on Children’s Food Literacy: The Mediating Role of Parent–Child Relationship and Learning Motivation.** *Nutrients* 2024, **16:**2564.

137. Yoshii E, Akamatsu R, Ishihara Y, Izumi B: **Impact of a school-based cooking programme on home cooking participation in Japan.** *Health Education Journal* 2021, **80:**375-386.

138. Zahr R, Sibeko L: **Influence of a School-Based Cooking Course on Students' Food Preferences, Cooking Skills, and Confidence.** *Can J Diet Pract Res* 2017, **78:**37-41.

139. Zarnowiecki D, Sinn N, Petkov J, Dollman J: **Parental nutrition knowledge and attitudes as predictors of 5–6-year-old children's healthy food knowledge.** *Public Health Nutrition* 2012, **15:**1284-1290.
